# A Fox2-Dependent Fatty Acid ß-Oxidation Pathway Coexists Both in Peroxisomes and Mitochondria of the Ascomycete Yeast *Candida lusitaniae*

**DOI:** 10.1371/journal.pone.0114531

**Published:** 2014-12-08

**Authors:** Frédéric Gabriel, Isabelle Accoceberry, Jean-Jacques Bessoule, Bénédicte Salin, Marine Lucas-Guérin, Stephen Manon, Karine Dementhon, Thierry Noël

**Affiliations:** 1 Univ. Bordeaux, Microbiologie Fondamentale et Pathogénicité, UMR 5234, F-33000 Bordeaux, France; 2 CNRS, Microbiologie Fondamentale et Pathogénicité, UMR 5234, F-33000 Bordeaux, France; 3 Univ. Bordeaux, Laboratoire de Biogenèse Membranaire, UMR 5200, F-33000 Bordeaux, France; 4 CNRS, Laboratoire de Biogenèse Membranaire, UMR 5200, F-33000 Bordeaux, France; 5 Univ. Bordeaux, Institut de Biochimie et Génétique Cellulaires, UMR 5095, F-33000 Bordeaux, France; 6 CNRS, Institut de Biochimie et Génétique Cellulaires, UMR 5095, F-33000 Bordeaux, France; INRA, France

## Abstract

It is generally admitted that the ascomycete yeasts of the subphylum *Saccharomycotina* possess a single fatty acid ß-oxidation pathway located exclusively in peroxisomes, and that they lost mitochondrial ß-oxidation early during evolution. In this work, we showed that mutants of the opportunistic pathogenic yeast *Candida lusitaniae* which lack the multifunctional enzyme Fox2p, a key enzyme of the ß-oxidation pathway, were still able to grow on fatty acids as the sole carbon source, suggesting that *C. lusitaniae* harbored an alternative pathway for fatty acid catabolism. By assaying ^14^C_α_-palmitoyl-CoA consumption, we demonstrated that fatty acid catabolism takes place in both peroxisomal and mitochondrial subcellular fractions. We then observed that a *fox2*Δ null mutant was unable to catabolize fatty acids in the mitochondrial fraction, thus indicating that the mitochondrial pathway was Fox2p-dependent. This finding was confirmed by the immunodetection of Fox2p in protein extracts obtained from purified peroxisomal and mitochondrial fractions. Finally, immunoelectron microscopy provided evidence that Fox2p was localized in both peroxisomes and mitochondria. This work constitutes the first demonstration of the existence of a Fox2p-dependent mitochondrial β-oxidation pathway in an ascomycetous yeast, *C. lusitaniae*. It also points to the existence of an alternative fatty acid catabolism pathway, probably located in peroxisomes, and functioning in a Fox2p-independent manner.

## Introduction

**Figure 1:**
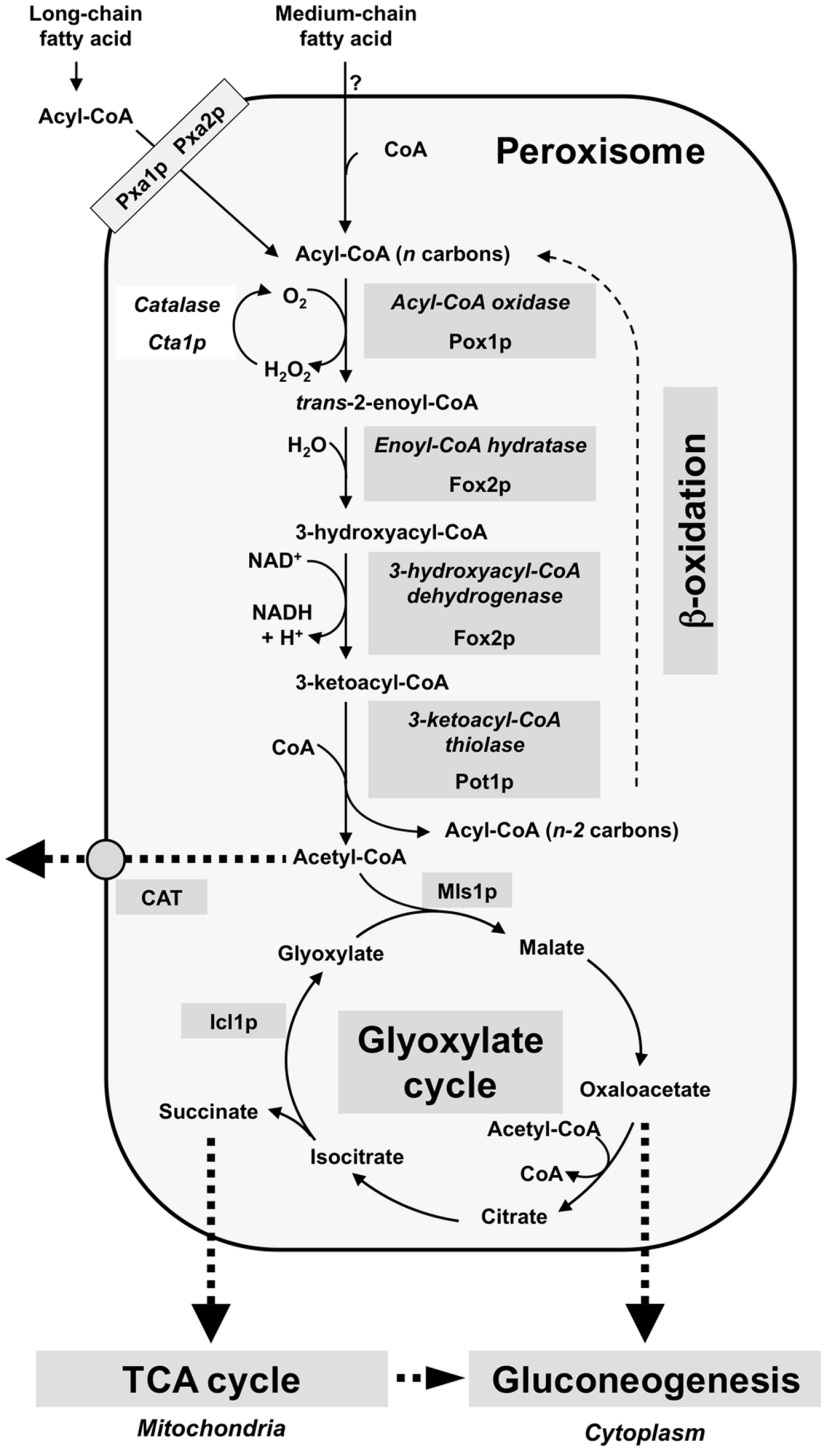
Fatty acid catabolism in ascomycetous yeasts. Model adapted from [3], [4]. The β-oxidation pathway is exclusively peroxisomal. The localization of the specific enzymes of the glyoxylate cycle (Icl1p and Mls1p) is susceptible to variations according to the species (i. e. Icl1p is peroxisomal in *C. albicans* and cytoplasmic in *S. cerevisiae*). Mls1p: Malate synthase. CAT: carnitine acetyl-transferase.

Lipid metabolism in fungal pathogenesis has recently attracted growing interest because of its possible multiple implications during host-pathogen interactions [1], [2]. One particular issue addresses the role of the catabolism of fatty acids (FA) as a source of nutrient when a fungal pathogen invades the host [2], [3]. In the ascomycetous yeasts *Saccharomyces cerevisiae*, *Yarrowia lipolytica*, and *Candida tropicalis*, the β-oxidation of both long- and short-chain FA is exclusively localized in the peroxisomes [4]–[9]. In *S. cerevisiae*, the long chain FA are transported across the peroxisomal membrane via a heterodimeric ABC transporter Pxa1p-Pxa2p [4], [10], [11], while the medium-chain FA are thought to be imported as free FA [4], [12]. Fatty acids are activated to fatty acyl-coenzyme A esters (FA-CoA) by acyl-CoA synthetases, and then shortened between carbon 2 and 3 through the β-oxidation spiral, yielding acetyl-CoA in four steps (**Fig. 1**): **i/**an acyl–CoA–oxidase (Pox1p *alias* Fox1p) converts FA-CoA into *trans*-2-enoyl-CoA and transfers electrons directly to oxygen generating H_2_O_2_ [4], which is detoxified by the Cta1p catalase [13]; **ii/and iii/**a multifunctional enzyme (Fox2p), which has both enoyl-CoA hydratase and 3-hydroxyacyl-CoA dehydrogenase activities, converts *trans*-2-enoyl-CoA into 3-ketoacyl-CoA [14], [15]; **iv**/a 3-ketoacyl-CoA thiolase (Pot1p *alias* Fox3p) converts 3-ketoacyl-CoA into acetyl-CoA and FA-CoA, shortened by two carbon units, which then can undergo an additional β-oxidation cycle. Acetyl units may integrate the glyoxylate cycle [16], or be exported outside the peroxisome by the carnitine acetyl transferase shuttle system, or be exported as citrate or malate [3].

There are very few examples of β-oxidation systems located outside the peroxisomes in the fungal kingdom. A mitochondrial location was described in some evolutionary divergent species including ascomycetous molds and basidiomycetous yeasts [17]–[21]. Nevertheless, a common way of thinking is to consider that mitochondrial ß-oxidation was lost early during the evolution of the ascomycetous yeasts [9], [22]. In this work, we demonstrate that several pathways of catabolism of FA coexist in the ascomycetous yeast *Candida lusitaniae*, of which one is a Fox2p-dependent ß-oxidation pathway taking place both in the peroxisomes and in the mitochondria.

*Candida lusitaniae* (teleomorph *Clavispora lusitaniae*) is an opportunistic pathogenic yeast belonging to the CTG clade of *Saccharomycotina*, i.e. yeasts that translate CTG as serine instead of leucine. It can be responsible for infections in immune deficient patients, especially those suffering from hematological malignancies, and in premature neonates [23]. Although its frequency in invasive candidiasis is low, *C. lusitaniae* infections are feared because they are sometimes associated with resistance to antifungal treatment, notably amphotericin B [23]. This yeast is also an interesting alternative laboratory model for biological studies, because it has a sexual reproduction [24] controllable *in vitro* [25], and its haploid genome is easily amenable to reverse genetic studies owing to different molecular tools [26]–[28].

Phagocytic cells are the primary line of defense against *Candida* infections. However, some species like *Candida albicans* and *Candida glabrata* are able to resist macrophage phagolysis and to escape from the phagocytic cell [16], [29], [30]. Upon phagocytosis by the macrophages, *C. albicans* reprograms its metabolism to down-regulate glycolysis and to up-regulate genes involved in the peroxisomal metabolism. The key enzymes of the glyoxylate cycle, isocitrate lyase and malate synthase, are thus strongly induced along with the FA β-oxidation genes [31], [32]. Even if the glyoxylate cycle may be considered as a virulence factor in some human and plant pathogenic fungi, such as *C. albicans* [16] and *Magnaporthe grisea* [33], the role of β-oxidation during pathogenesis is controversial [34], [35].

The present work was initiated to understand better the relationship between the β-oxidation of FA and the glyoxylate cycle in *C. lusitaniae*, with the goal of identifying the source of acetyl-CoA that feeds the glyoxylate cycle. For that, we constructed different null-mutants in *C. lusitaniae*: *icl1*Δ, *fox2*Δ, and *pxa1*Δ, lacking the isocitrate lyase, the multifunctional β-oxidation protein, and a protein responsible for the peroxisomal long chain FA uptake, respectively. Growth assays and palmitoyl-CoA catabolism assays revealed that the catabolism of long-chain FA-CoA was still functional in a *C. lusitaniae fox2Δ* mutant. Subcellular fractionation, protein immunoblot and immunoelectron microscopy enabled us to demonstrate that *C. lusitaniae* harbors a peroxisomal and a mitochondrial Fox2p-dependent β-oxidation pathway, and hosts an additional peroxisomal Fox2p-independent pathway that allows a *fox2*Δ mutant to use FA as the sole carbon source.

## Results

### A *C. lusitaniae fox2*Δ mutant can grow on medium-chain and long-chain saturated FA as sole carbon source, whereas an *icl1*Δ mutant cannot

The unique genes *ICL1* encoding the isocitrate lyase, *FOX2* encoding the multifunctional protein of β-oxidation, and *PXA1* encoding part of an ABC transporter responsible for peroxisomal long chain FA uptake, were identified in the genome of *C. lusitaniae* with a BLAST analysis [36] using as query the orthologous proteins of *C. albicans*. Orthology was determined after considering several parameters including homology, synteny and identification of the domains characteristic of each protein family (**S1 File**). No paralogous gene could be detected for *ICL1*, *FOX2* and *PXA1* in the genome of *C. lusitaniae*. The null mutants *icl1*Δ, *pxa1*Δ and *fox2*Δ, and the relevant reintegrant strains bearing a reconstituted functional allele, were then constructed in *C. lusitaniae* using a *URA3*-blaster strategy [28]. In the case of *FOX2*, two *fox2*Δ null-mutants were constructed in independent ways, one bearing a deletion of 1543 bp, and the other a deletion of 3012 bp. All mutants were characterized at the molecular level by PCR, Southern blot and nucleotide sequencing of the target locus (*see* **S1 Figure** for the design and the characterization by Southern blot of a *fox2*Δ mutant strain and of the corresponding reintegrant strain). *S. cerevisiae* and *C. albicans* mutants with defect in peroxisomal β-oxidation (i.e. *fox2*Δ) or in glyoxylate cycle (i.e. *icl1*Δ) are not able to utilize FA as the sole carbon source [4], [34], [35]. We tested the ability of the *C. lusitaniae icl1*Δ and *fox2*Δ mutants to grow on minimal medium (YNB agar) containing either glucose, glycerol, acetate, saturated short-chain FA (C10:0), saturated medium-chain FA (C12:0, C14:0), saturated long-chain FA (C16:0, C18:0), or unsaturated long-chain FA (C18:1, C22:1), as the exclusive carbon source (**Table 1**). All strains grew equally on medium containing glucose and none of them was able to grow on unsupplemented YNB agar (see the drop test in **S2 Figure**). The wild type strain 6936 was able to grow on all the carbon sources, except on the medium containing C10:0. Additional growth tests were performed using YNB agar supplemented with both C10:0 and glucose (0.1%, 1% and 2%). The growth failure of the wild type strain on these media suggested that the C10:0 itself or a by-product of its catabolism was toxic for the *C. lusitaniae* cells (data not shown). The *C. lusitaniae icl1*Δ mutant was unable to assimilate acetate and FA, as previously described for *C. albicans* [16], [35], but was able to grow on glycerol, whereas the *C. albicans* mutant did not [35]. Overall, this confirmed that the glyoxylate cycle is an essential pathway for the utilization of non-fermentable carbon sources in *Candida* yeasts. Surprisingly, the deletion of *FOX2* did not abolish growth on medium-chain and long-chain saturated FA in *C. lusitaniae* (see **S2 Figure** for an example of drop test on C16:0 YNB agar). The two *fox2*Δ mutants also grew on acetate and glycerol. Growth ability of the *fox2*Δ mutants was partially impaired in the presence of unsaturated fatty acids C18:1 and C22:1 (**S2 Figure**). However, growth was fully restored when YNB agar was supplemented with both unsaturated fatty acid and glucose (**S2 Figure**), indicating that the growth defect resulted from a default of assimilation rather than from a toxic effect of the fatty acid itself. An additional control was performed by measuring and comparing growth of different strains and mutants of *C. lusitaniae* and *S. cerevisiae* in liquid YNB supplemented with oleic acid (**S3 Figure**). This test showed that, when used as single carbon source, oleic acid was more efficiently assimilated by *C. lusitaniae* than by *S. cerevisiae*, and that a *fox2* mutant of *C. lusitaniae* was able to use oleic acid whereas a *fox2* mutant of *S. cerevisiae* did not. However, the *fox2* mutant of *C. lusitaniae* had a growth on oleic acid reduced by about 50% when compared to the wild type strain. Overall, our results strongly suggest the existence of a Fox2p-independent FA catabolism pathway in *C. lusitaniae*. This alternate pathway could have a lower affinity for unsaturated fatty acids (oleic and erucic acids) and medium-chain fatty acid (lauric acid) than the normal ß-oxidation pathway, as suggested by the reduced growth of the mutant strains having a *fox2* deletion on these fatty acids.

**Table 1:** Growth characteristics of mutant, reintegrant, and wild-type strains on different carbon sources at 30°C.

YNB agar	*icl1*Δ	*ICL1Re*	*fox2*Δ	*FOX2Re*	*pxa1*Δ	*fox2*Δ, *pxa1*Δ	*6936*
Unsupplemented	-	-	-	-	-	-	-
Glucose	+++++	+++++	+++++	+++++	+++++	+++++	+++++
Acetate	-	+++++	+++++	+++++	+++++	+++++	+++++
Glycerol	+++++	+++++	+++++	+++++	+++++	+++++	+++++
Capric acid (C10:0)	-	-	-	-	-	-	-
Lauric acid (C12:0)	-	+++++	+++	+++++	+++++	+++	+++++
Myristic acid (C14:0)	-	+++++	+++++	+++++	+++++	+++++	+++++
Palmitic acid (C16:0)	-	+++++	+++++	+++++	+++++	+++++	+++++
Stearic acid (C18:0)	-	+++++	+++++	+++++	+++++	+++++	+++++
Oleic acid (C18:1)	-	+++++	+++	+++++	+++++	+++	+++++
Erucic acid (C22:1)	-	+++++	+++	+++++	+++++	+++	+++++

Cells were pregrown to mid-log phase in YPD, washed, resuspended in water to an OD_600_ of 0.5 (about 8.5×10^6^ cells/ml), and serially diluted fivefold. Drops of 7 µl of each dilution were deposited onto solid minimum media (YNB), containing 2% of either glucose, acetate, glycerol, or fatty acid as the sole carbon source. Growth was observed after 3 days (glucose) or 5 days (acetate, glycerol, fatty acids) of incubation at 30°C. Growth on the first dilution (1∶5) was noted ‘+’, on the second dilution (1∶5^2^) was noted ‘++’, on the third dilution (1∶5^3^) was noted ‘+++’, on the fourth dilution (1∶5^4^) was noted ‘++++’, on the fifth dilution (1∶5^5^) was noted ‘+++++’, and no growth was noted ‘−’.

### A *C. lusitaniae pxa1*Δ mutant is able to grow on long-chain saturated FA as the sole carbon source

**Figure 2:**
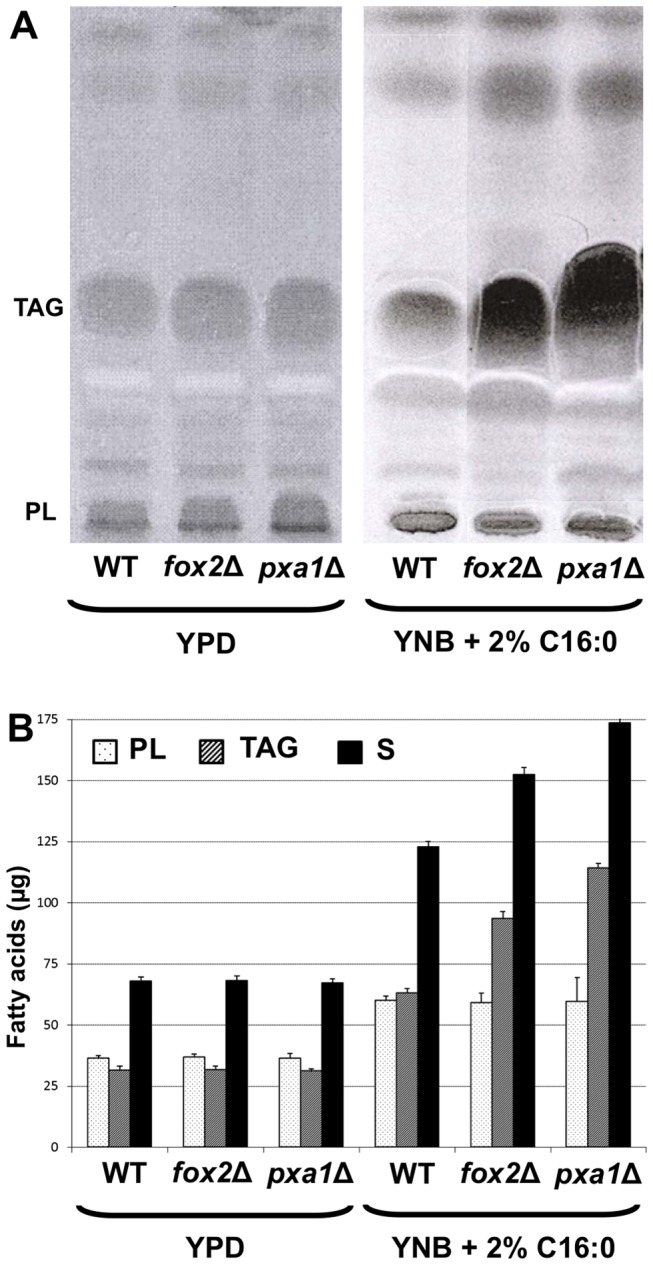
Neutral lipid composition of the wild-type, *fox2*Δ and *pxa1*Δ strains. (A) Thin-layer chromatography (TLC) of lipid extracts obtained from the wild-type, *fox2*Δ and *pxa1*Δ strains after growth on YPD or YNB +2% C16:0. The silica gel plates were observed under UV light after spraying of primuline. PL: phospholipids, TAG: tri-acyl-glycerol. (B) Amount of FA contained in PL (light gray) and TAG (dark gray) fractions purified by TLC and quantified by gas-chromatography. C17:0 was used as standard. The sum of the PL and TAG fractions are represented with black bars. Error bars represent SE.

In *S. cerevisiae*, the disruption of *PXA1*, encoding a long-chain FA peroxisomal import protein, results in impaired growth on oleic acid and reduced ability to oxidize oleate [11]. Disruption of *PXA1* in *C. lusitaniae* had no effect on FA utilization. The *pxa1*Δ mutant was able to grow on C22:1, C18:1, C18:0, C16:0, C14:0 and C12:0 as the sole carbon source (**Table 1**). One possibility was that long-chain FA could still penetrate peroxisomes using another transporter, as recently demonstrated in the filamentous fungus *P. anserina* [20]. Another possibility was that the transport of FA across the peroxisomal membrane was really impaired in the *pxa1*Δ mutant, thus leading to lipid – especially triacylglycerols (TAG) – accumulation within the cell [37]. We therefore analyzed neutral lipid contents of *fox2*Δ, *pxa1*Δ and wild-type strains. The amount of phospholipids (PL) and TAG extracted from the *fox2*Δ and *pxa1*Δ mutant strains after growth on YPD were not significantly different from the wild-type strain (**Fig. 2**). However, growth on YNB +2% C16:0 yielded a significant augmentation of TAG and PL amounts (x 2, ×2.9 and ×3.7, respectively for the wild-type strain, *fox2*Δ and *pxa1*Δ strains). TAG accumulation in the *fox2*Δ cells can be the consequence of the ß-oxidation default, which slow down the intracellular lipid trafficking and possibly leads to the storage of excess unmetabolized FA. Even more remarkable was the amount of TAG extracted from the *pxa1*Δ mutant strain, which was significantly higher than in the wild-type strain (p<0.05). This suggested that transport of FA to peroxisomes was impaired in the *pxa1*Δ mutant, and that a functional FA catabolic pathway could occur in a subcellular compartment differing from the peroxisomes, allowing growth of the mutant on long chain FA. To test this hypothesis, we constructed the double mutant strain *pxa1*Δ, *fox2*Δ and determine its growth abilities. The double mutant was able to use fatty acids as sole carbon source, as did the simple mutant *fox2*Δ (**Table 1**). This result strengthened the hypothesis that a fatty acid oxidation pathway independent from Fox2p could take place in *C. lusitaniae*, possibly outside the peroxisome.

### Long-chain FA-CoA catabolism is still functional in the *C. lusitaniae fox2*Δ and *pxa1*Δ mutants

**Figure 3:**
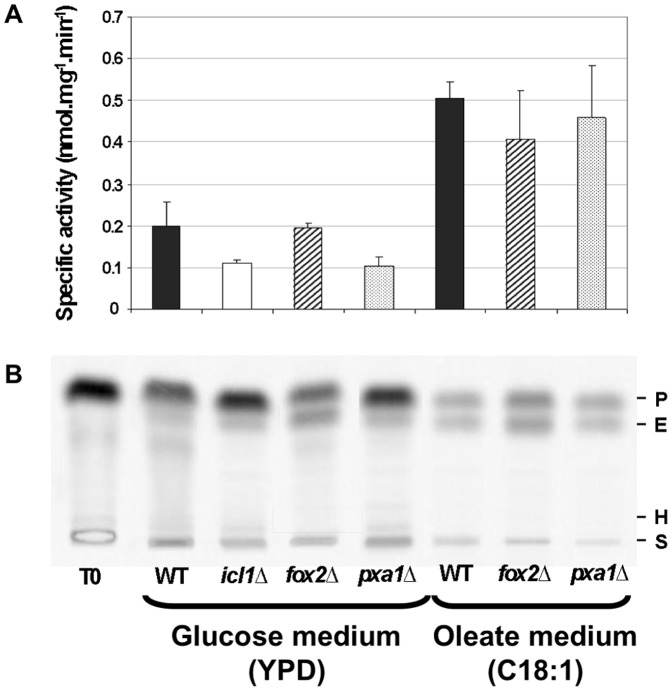
Consumption of ^14^C_α_-palmitoyl-CoA by cellular crude extracts of the *C. lusitaniae icl1*Δ, *fox2*Δ, *pxa1*Δ and wild-type strains. (A) ^14^C_α_-palmitoyl-CoA consumption rates after 15 min at 37°C by crude extracts of the mutant and wild-type strains after analysis of the chromatograms using ImageQuant software. Error bars represent SE. (B) Corresponding chromatograms. T0: Amount of ^14^C_α_-palmitoyl-CoA initially present in the reaction mix. WT: wild-type strain. P: ^14^C_α_-palmitoyl-CoA. E: ^14^C_α_-hexadecenoyl-CoA. H: ^14^C_α_-3-hydroxyhexadecenoyl-CoA. S: start.

As the *C. lusitaniae fox2*Δ and *pxa1*Δ mutants are able to assimilate long-chain FA, we measured the consumption of ^14^C_α_-palmitoyl-CoA by crude cellular extracts. There was no significant difference between the specific activities observed for the *icl1*Δ, *fox2*Δ, *pxa1*Δ and wild-type strains grown on glucose medium (**Fig. 3**). The *fox2*Δ, *pxa1*Δ and wild-type strains were then cultivated in C18:1 medium to induce FA β-oxidation [38]. As expected, the specific activities were increased 2 to 3 fold after induction, but no significant difference could be detected between the mutant and the wild-type strains.

### Long-chain FA-CoA catabolism is functional in the peroxisomal fraction of the *C. lusitaniae fox2*Δ mutant

**Figure 4:**
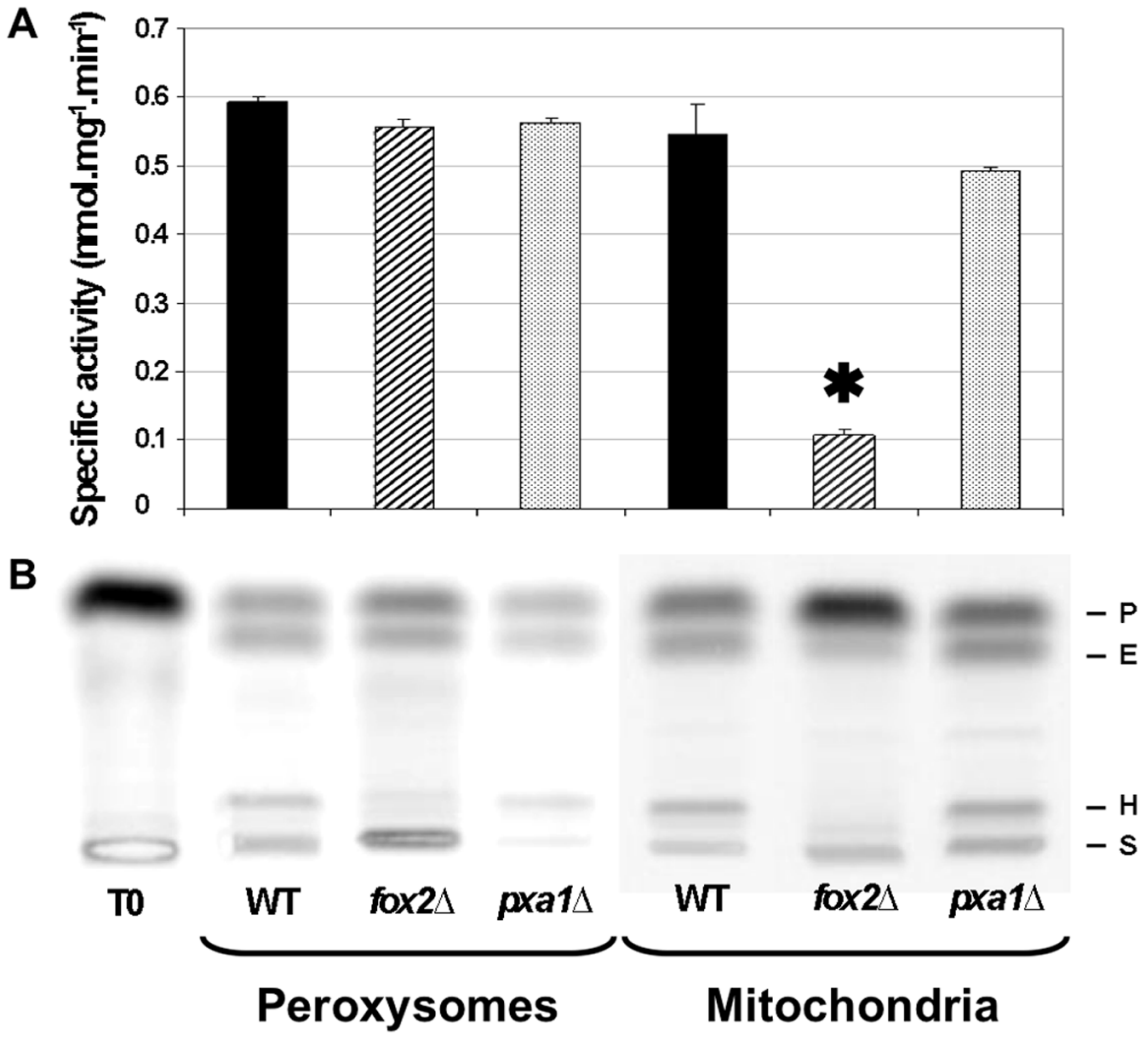
Consumption of ^14^C_α_-palmitoyl-CoA by peroxisomal and mitochondrial fractions of the *C. lusitaniae fox2*Δ, *pxa1*Δ and wild-type strains. (A) ^14^C_α_-palmitoyl-CoA consumption rates after 15 min at 37°C by peroxisomal and mitochondrial fractions of the mutant and wild-type strains after analysis of the chromatograms using ImageQuant software. The activity observed in the mitochondrial fraction of *fox2*Δ and wild-type strains were compared using a Student's t-test (*, p = 0.016). Error bars represent SE. (B) Corresponding chromatograms. T0: Amount of ^14^C_α_-palmitoyl-CoA initially present in the reaction mix. WT: wild-type strain. P: ^14^C_α_-palmitoyl-CoA. E: ^14^C_α_-hexadecenoyl-CoA. H: ^14^C_α_-3-hydroxyhexadecenoyl-CoA. S: start.

Mitochondrial and peroxisomal fractions were obtained from the mutant and the wild-type strains using a discontinuous sucrose gradient. The purity of each organelle fraction was estimated by assaying in parallel catalase and cytochrome *c* oxidase which were considered as enzymatic markers specific to the peroxisomal and mitochondrial fraction, respectively (**Table 2**). The peroxisomal fraction of the *fox2*Δ, *pxa1*Δ and wild-type strains exhibited a high specific activity for catalase (ranging from 404 to 658 U/mg of protein) and a high ratio of catalase/cytochrome *c* oxidase activities (from 918 to 1834). The mitochondrial fraction exhibited nearly a 20-fold lower ratio. The contamination of the mitochondrial fraction by peroxisomes did not exceed 20% (for example, for the wild-type strain, the ratio of the catalase activity of the mitochondrial fraction versus the catalase activity of the peroxisomal fraction was 0.21). The consumption of ^14^C_α_-palmitoyl-CoA was then assayed with the protein extracts obtained from each fraction (**Fig. 4**). Surprisingly, the consumptions of ^14^C_α_-palmitoyl-CoA by peroxisomal fractions of the *fox2*Δ, *pxa1*Δ and the wild-type strains were similar. This result supported the idea that the peroxisomes of *C. lusitaniae* harbored a Fox2p-independent FA-CoA catabolism pathway.

**Table 2:** Purification of peroxisomes and mitochondria from cells grown in induction medium.

Strain	Fraction^a^	Catalase Sp activity (U/mg)		Cytochrome c oxidase Sp activity (U/mg)		catalase/oxidase ratio^b^	mitochondria/peroxisomes catalase ratio^c^
Wild-type	peroxisomes	646	+/− 246	0.352	+/− 0.099	1834	0.21
	mitochondria	136	+/− 68	1.492	+/− 0.444	91	
*fox2*Δ	peroxisomes	404	+/− 32	0.440	+/− 0.036	918	0.09
	mitochondria	37	+/− 17	0.764	+/− 0.018	49	
*pxa1*Δ	peroxisomes	658	+/− 258	0.360	+/− 0.104	1828	0.22
	mitochondria	145	+/− 82	1.235	+/− 0.333	117	

Sp activity: specific activity

a 10 µg of proteins

b Catalase specific activity was divided by cytochrome c oxidase specific activity

c Catalase specific activity in mitochondrial fraction was divided by catalase specific activity in peroxisomal fraction

### Long-chain FA can be catabolized in the mitochondrial fraction of the *C. lusitaniae* wild-type strain, but not in the mitochondrial fraction of the *fox2*Δ mutant

**Figure 5:**
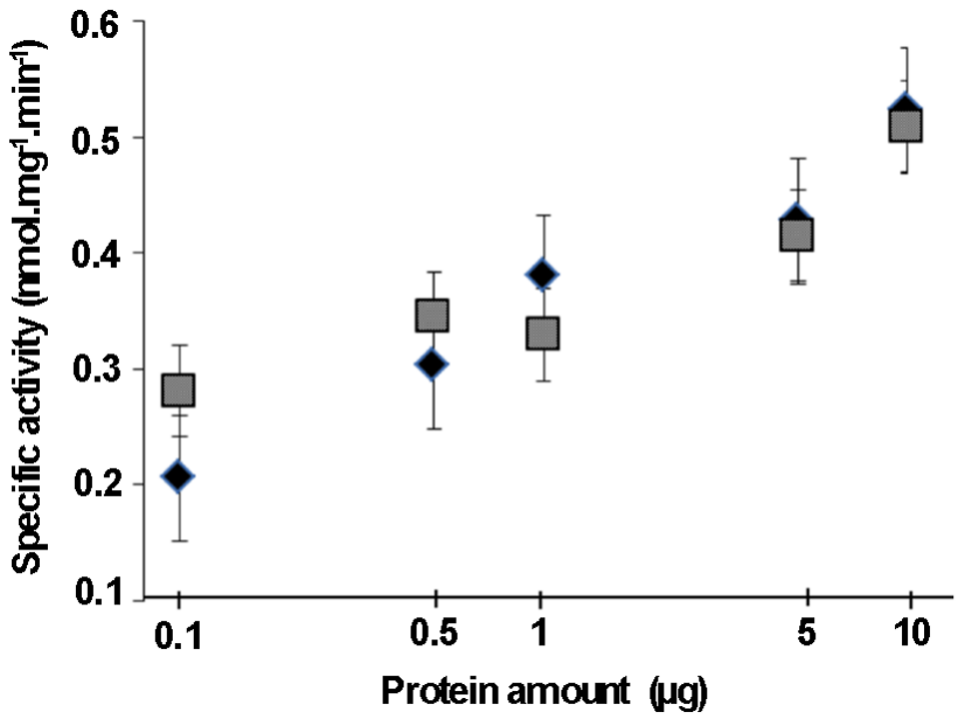
Consumption of ^14^C_α_-palmitoyl-CoA by different quantity of proteins from the peroxisomal and mitochondrial fractions of the *C. lusitaniae* wild-type strain. ^14^C_α_-palmitoyl-CoA consumption rates after 2 min at 37°C by peroxisomal (diamonds) and mitochondrial (squares) fractions of the wild-type strains. Activity is expressed as nmol of ^14^C_α_-palmitoyl-CoA consumed per min after analysis of the chromatograms using ImageQuant software. Error bars represent SE.

Unexpectedly, a consumption of ^14^C_α_-palmitoyl-CoA was observed in the mitochondrial fractions of the *pxa1*Δ and wild-type strains (**Fig. 4**). The specific activities were similar to those measured in the peroxisomal fractions. We further demonstrated that the FA catabolism in each of the mitochondrial and peroxisomal fractions increased when using from 0.1 µg to 10 µg of proteins to perform the assay (**Fig. 5**). In this range, specific activities were similar in both the mitochondrial and the peroxisomal fractions of the wild type strain. Surprisingly, the ^14^C_α_-palmitoyl-CoA catabolism was significantly decreased in the mitochondrial fractions of the *fox2*Δ mutant (p<0.05), and the residual activity (nearly 20%) fitted the percentage of the peroxisomal contamination previously estimated by the catalase activity (**Fig. 4**). These results strongly supported the existence of a Fox2p-dependent catabolism of FA-CoA in the mitochondria of *C. lusitaniae*.

### Fox2p is localized in both peroxisomal and mitochondrial protein fractions of *C. lusitaniae*

**Figure 6:**
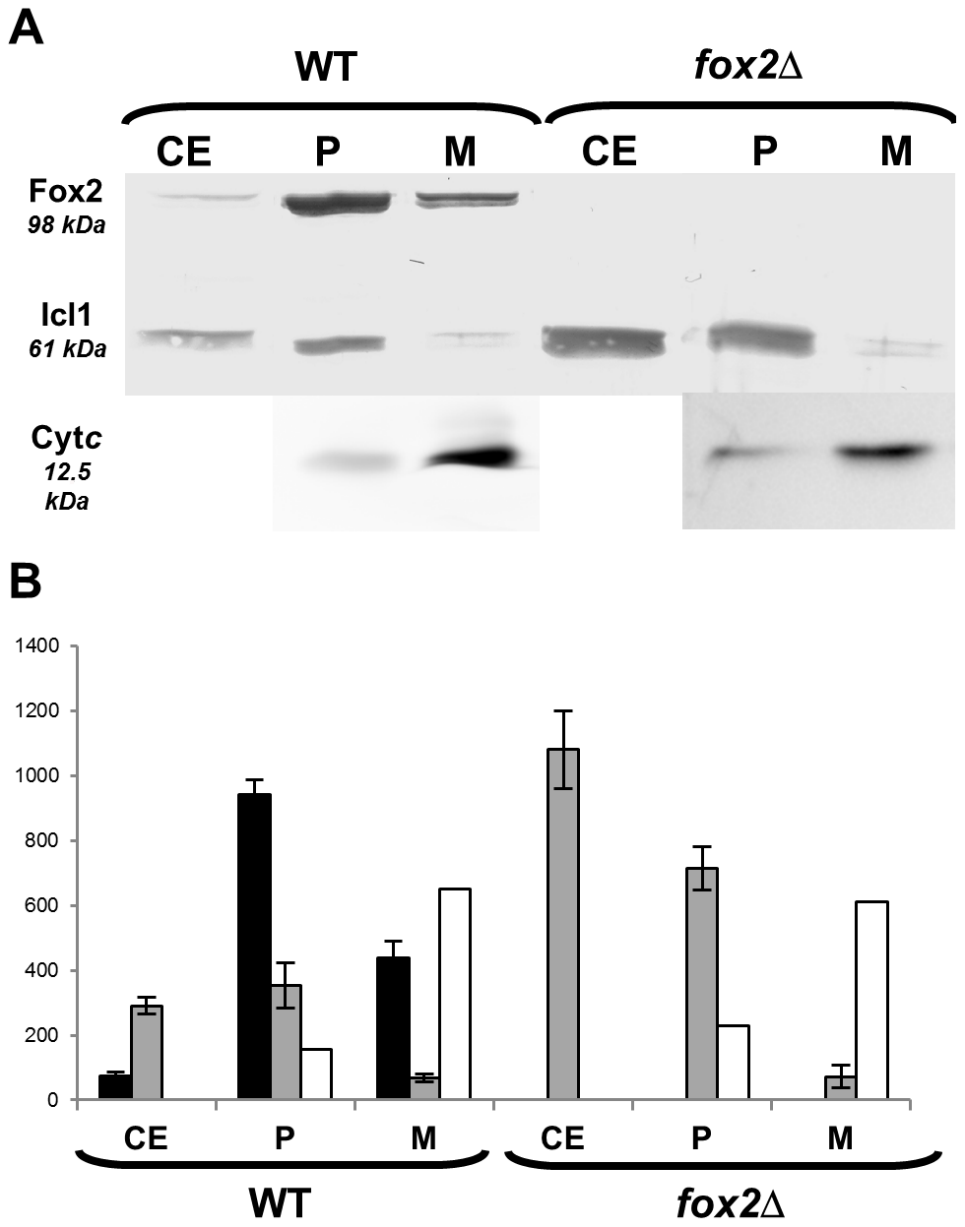
Immunodetection of Fox2p, Icl1p and cytochrome c by Western-blot in the *C. lusitaniae fox2*Δ and wild-type strains. (A) Thirty µg of proteins of the crude extracts and 10 µg of proteins of the peroxisomal and mitochondrial fractions were separated by SDS-PAGE. (B) Signal integration expressed in relative intensity using Quantity One software. Relative amounts of Fox2p are represented with black bars, Icl1p with grey bars, and Cyt*c* with white bars. Error bars represent SE. Cyt*c*: cytochrome *c*, WT: wild type, CE: crude extract, P: peroxisomal fraction, M: mitochondrial fraction.

Western-blotting analysis was performed using polyclonal anti-Fox2p and anti-Icl1p antibodies on crude extracts, peroxisomal or mitochondrial fractions of the *fox2*Δ mutant and of the wild-type strains. Antibodies anti-Cytochrome *c* (Cyt*c*) was also used as a control. In *C. albicans*, it was shown that Icl1p, a key enzyme of the glyoxylate cycle, was localized in peroxisomes [39]. We confirmed that Icl1p was present in the peroxisomal fractions of both the *C. lusitaniae fox2*Δ mutant and wild-type strains. A faint signal, not exceeding 10 to 20% of the amount of Icl1p present in the peroxisomal fraction, was also detected in the mitochondrial fraction (**Fig. 6**). This corresponded to the level of contamination of the mitochondrial fraction by peroxisomes that we had already estimated using catalase assays. Reciprocally, a slight contamination of the peroxisomal fraction by mitochondrial proteins was revealed with the anti-Cyt*c* antibody. On the other hand, and as expected, Fox2p was undetectable in the *fox2*Δ mutant. Interestingly, Fox2p was detected in both peroxisomal and mitochondrial fractions of the wild type strain (**Fig. 6**). The amount of Fox2p detected in the mitochondrial fraction was nearly 50% of that of the peroxisomal fraction.

### Immunoelectron microscopy localization of Fox2p both in peroxisomes and mitochondria of *C. lusitaniae*

**Figure 7:**
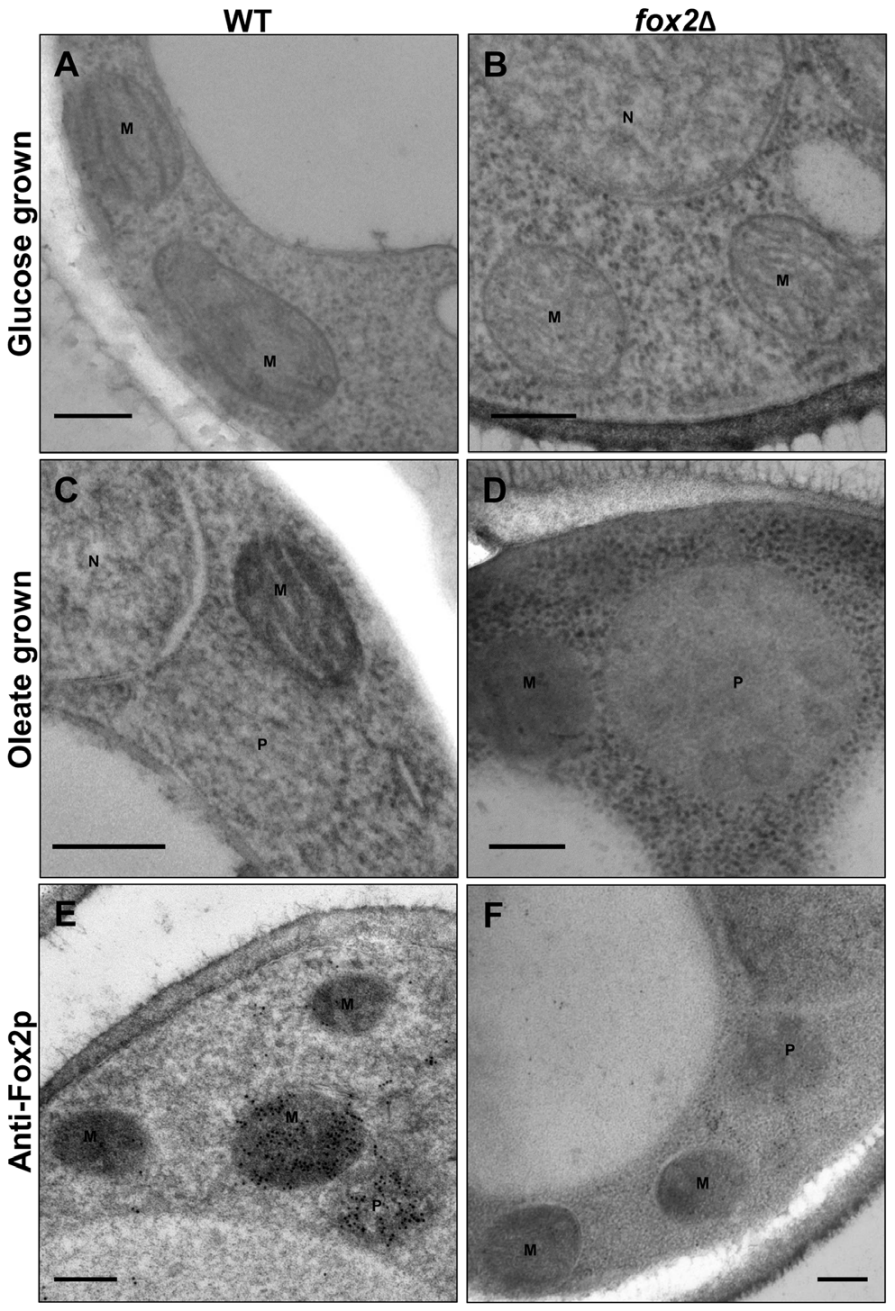
Characterization of the wild-type and *fox2*Δ strains by electron and immunoelectron microscopy. Glucose-grown cells (A and B) or oleate-induced cells (C to F) of wild-type (A, C, and E) or *fox2*Δ (B, D, and F) strains were fixed and prepared for electron microscopy (A to D) or immunoelectron microscopy (E and F). In E and F, cryosections were incubated with a polyclonal hen primary antibody directed against *C. lusitaniae* Fox2p and antibodies were revealed with immunogold particles conjugated to anti-hen antibodies. P, peroxisome, M, mitochondrion, N, nucleus. Bars, 200 nm.

Apparent ultrastructural changes of both peroxisomes and mitochondria were observed in *C. lusitaniae* cells according to the culture medium. When compared to YPD cultures, yeast cells grown on oleic acid contained more abundant peroxisomes; surprisingly, their size was approximately two-fold greater in the *fox2*Δ cells than in the wild-type cells (**Fig. 7**, **Table 3**). Moreover, the *fox2*Δ peroxisomes were characterized by the presence of several round-shaped inclusions (**Fig. 7D**). The number and size of mitochondria sections per cell section were similar for the wild type and *fox2*Δ strains whatever the growth medium used (**Table 3**). Interestingly, the apparent density of mitochondria was greater in wild-type than in *fox2*Δ cells, and greater in wild type cells grown in oleate compared to YPD. Immunogold labeling with rabbit polyclonal antibody anti-Icl1p confirmed that isocitrate lyase was localized in peroxisomes in *C. lusitaniae* (data not shown). As expected, a hen polyclonal antibody specifically directed against the *C. lusitaniae* Fox2p allowed detection of Fox2p in peroxisomes, but also in mitochondria. Gold label in mitochondria was heterogeneous but very strong in some mitochondrial sections (**Fig. 7E**). No specific labeling could be detected in peroxisomes and mitochondria of the *fox2*Δ mutant (**Fig. 7F**).

**Table 3:** Number and size of peroxisomes and number of mitochondria sections observed in *Candida lusitaniae* cells by electron microscopy.

		wild-type		*fox2*Δ	
		glucose-grown	oleate-grown	glucose-grown	oleate-grown
**Peroxisomes**	**number per cell section** a	0.08±0.3	1±0.7	0.02±0.3	0.8±0.5
	**diameter (nm)**	206±11.5	245±41	198±22	462±96
**Mitochondria**	**number per cell section** a	4±1.4	3.8±2	3.4±1.3	4.3±1.2

a Only cell sections containing observable nucleus and vacuole were considered.

## Discussion

In this work, we demonstrated the existence of a mitochondrial β-oxidation pathway in an ascomycetous yeast, *C. lusitaniae*, and of an alternative peroxisomal Fox2p-independent pathway of fatty acid catabolism. So far, it has been shown that the ascomycete yeasts, such as *S. cerevisiae*, *Yarrowia lipolytica*, and *C. tropicalis*, possessed only a peroxisomal β-oxidation pathway [4], [5], [7], and it has been extrapolated and widely admitted that all the species of the subphylum *Saccharomycotina* lacked mitochondrial ß-oxidation [8]. More recently, an *in silico* analysis of diverse fungal genomes failed to identify complete sets of enzymes that could be involved in non-peroxisomal β-oxidation in ascomycete yeasts, and it was logically concluded that this pathway was probably lost very early during the evolution of *Saccharomycotina* [9], [22]. However, a mitochondrial β-oxidation pathway was suspected for a long time in the ascomycetous yeast *C. tropicalis*, when both acyl-CoA oxidase and acyl-CoA dehydrogenase, which were thought to be linked to the peroxisomal and the mitochondrial β-oxidation pathway respectively, were characterized at the biochemical level [40]. However, it was argued that acyl-CoA dehydrogenases were not specific to mitochondrial β-oxidation, as they can also be involved in the degradation of valine, leucine, and isoleucine, as recently confirmed in *Aspergillus nidulans* [41]. It was then definitely considered that β-oxidation was exclusively located in the peroxisomes in *C. tropicali*s, because the four main enzymes of ß-oxidation, *i.e.* acyl-CoA oxidase, enoyl-CoA hydratase, hydroxyacyl-CoA dehydrogenase and 3-ketoacyl-CoA thiolase were localized in the same subcellular compartment as the typical peroxisomal enzymes catalase and isocitrate lyase [6]. However, not all fungi have lost extra-peroxisomal β-oxidation; it was recently clearly demonstrated that it occurred both in peroxisomes and in mitochondria of the ascomycete filamentous fungi *A. nidulans* [19] and *Podospora anserina* [20], and possibly in the basidiomycetous yeast *Sporidiobolus pararoseus* [21].

In this work, we first showed that a *fox2*Δ mutant of *C. lusitaniae*, defective for the multifunctional enzyme of ß-oxidation, and a *pxa1*Δ mutant, defective for the uptake transport of long chain fatty acids into peroxisomes, were both still able to use and catabolize fatty acids as the sole carbon source. This result led us to investigate the possible occurrence of an extraperoxisomal pathway of fatty acid catabolism in *C. lusitaniae*. Then we showed that ^14^C_α_-palmitoyl-CoA catabolism was equally effective when catalyzed by a protein extract obtained from either purified peroxisomes or from purified mitochondria of a wild type strain, but not from mitochondria of a *fox2*Δ mutant. This result not only strongly suggested that a mitochondrial pathway of fatty acid catabolism did exist in *C. lusitaniae*, but also that this pathway was Fox2p-dependent. Western blot analysis of protein extracts obtained from organelle fractions of *C. lusitaniae* allowed detection of Fox2p in both the peroxisomal and mitochondrial fractions. Finally, immunoelectron microscopy completed the demonstration that Fox2p was localized in both peroxisomes and mitochondria in *C. lusitaniae*. The presence of Fox2p in the mitochondria of *C. lusitaniae* constitutes a unique phenotype in eukaryotes, in which mitochondrial ß-oxidation generally relies on other specific enzymes. In filamentous fungi, e.g. *A. nidulans*, and in mammals for FA-CoA chain of 12 carbons and fewer, FA catabolism is mediated by four reactions involving the four individual enzymes acyl-CoA dehydrogenase, enoyl-CoA hydratase, 3-hydroxy-acyl-CoA dehydrogenase, and 3-keto-acyl-CoA thiolase [9], [18], [19]. In mammals, for degrading FA-CoA greater than 12 carbons, the last three steps of the reaction are catalyzed by a trifunctional protein associated with the inner mitochondrial membrane [42]. When analyzed *a posteriori*, the double localization of Fox2p in peroxisomes and mitochondria in *C. lusitaniae* was difficult to predict with bioinformatic tools, as only a characteristic type I peroxisomal targeting signal (GKL) is present at the C-terminal part of the protein [43]. However, the dual targeting of peroxisomal proteins and of mitochondrial proteins in the cell may be dependent on more complex and diverse mechanisms, such as differential splicing, the use of alternative start codons, the ribosomal read-through of stop codons, the presence of multiple competing targeting signals, or post-translational modifications [44], [45].

Our work also shows the existence of a palmitoyl-CoA catabolism in purified peroxisomes of the *fox2*Δ mutant of *C. lusitaniae*. This alternative Fox2p-independent peroxisomal pathway allows the *C. lusitaniae fox2*Δ mutant to grow and to assimilate the saturated FA C12:0, C14:0, C16:0, C18:0, whereas a *fox2*Δ mutant of *C. albicans* cannot [34], [35]. Interestingly, this alternative pathway fully compensates the Fox2p defect when fed with long-chain saturated fatty acids, but only partially with lauric acid, and with the unsaturated oleic and erucic acids. Fox2p is a multifunctional protein, which possesses three catalytic domains, one for enoyl-CoA hydratase and two for 3-hydroxyacyl-CoA dehydrogenase activities. One can imagine that these activities could also be harbored by independent proteins, as in the mitochondrial β-oxidation pathway of filamentous fungi. Alternatively, two other pathways are known to drive FA oxidation in eukaryotic cells. One alternative oxidation pathway, ω-oxidation, occurs in peroxisomes of mammalian cells [46], and has already been described in the yeast *C. tropicalis* [47], [48]. Through this pathway, the monocarboxylic acids are first transformed into dicarboxylic acids which then need a functional β-oxidation to be shortened. Accordingly, ω-oxidation cannot explain the breakdown of the COOH- extremity of ^14^C_α_-palmitoyl-CoA observed in the *fox2*Δ mutant. Another alternative oxidation pathway, α-oxidation, may be used for the shortening of FA that cannot directly undergo β-oxidation, because of the presence of a methyl group on the carbon in position 3 or of a hydroxyl group on the carbon in position 2. In that case, shortening of the FA occurs by the removal of a single carbon from the carboxyl end and requires four enzymatic steps [49], [50]. Both peroxisomes and mitochondria were reported to host α-oxidation [49]–[51], but to our knowledge, this pathway has only been described in mammals, where its deficiency may lead to Refsum's disease [52]. Further investigations are needed to determine if an α-oxidation-like pathway does exist in *C. lusitaniae* and if it can compensate a defect in ß-oxidation to allow a *fox2*Δ mutant to grow on FA as the sole carbon source.

It is worth noting that some studies on phylogeny and evolution of fungi are based on the fatty acid catabolism pathway because of the diversity of its subcellular location (peroxisomal and/or extraperoxisomal), and of the variability of key enzymes of the pathway (use of acyl-CoA dehydrogenase and/or acyl-CoA oxidase, use of multifunctional *versus* monofunctional enzymes) [9], [22]. The discovery of a mitochondrial and peroxisomal Fox2p-dependent FA oxidation pathway in an ascomycetous yeast, along with an additional peroxisomal Fox2p-independent pathway of catabolism that remains to be characterized, confirms that the catabolism of FA in fungi displays strong evolutionary divergence across different taxonomic groups [9], [22], and that it could be more complex than expected. For example, the acyl-CoA dehydrogenases are considered as specific markers of the non-peroxisomal ß-oxidation in fungi, while acyl-CoA oxidases are specific to the peroxisomal ß-oxidation. Surprisingly, acyl-CoA dehydrogenases are absent from the genome of *C. lusitaniae* [53], which suggests that the oxidation of acyl-CoA in the mitochondria is dependent on another family of dehydrogenases, or that it requires the close cooperation of the peroxisomal acyl-CoA oxidases. The hypothesis of a "hybrid" ß-oxidation pathway between peroxisomes and mitochondria has already been formulated from the *in silico* analysis of the genomes of some *Sordariomycetes*, the filamentous ascomycetes, for which it was speculated that the apparent lack of peroxisomal acyl-CoA oxidases could be compensated by mitochondrial acyl-CoA dehydrogenases [9]. The experimental results presented here, notably the localization of Fox2p in both the peroxisomes and mitochondria of *C. lusitaniae*, are convergent with the idea that a hybrid ß-oxidation could take place in both organelles.

Our work sheds a new light on the wide diversity of a ubiquitous metabolic pathway such as beta-oxidation in fungi, and invites us to reconsider the postulate according to which the mitochondrial beta-oxidation was lost in *Saccharomycotina*. Our goal is now to characterize the different pathways of FA catabolism in *C. lusitaniae*, to explore whether these pathways do exist in other *Candida* species, and to determine their contribution to the virulence of this clade of yeasts which includes important opportunistic pathogens.

## Materials and Methods

### Strains and media

The *C. lusitaniae* strains used in this study are listed in **S1 Table**. All the mutant strains were derived from the wild-type strain 6936 *MAT*a (Centraalbureau voor Schimmelcultures, Utrecht, The Nederlands). For some control experiments, we used the *S. cerevisiae* wild type strain BY4742 *MAT*α and the corresponding *fox2* mutant strain Y15080 *MAT*α, *ura3*Δ0, *leu2*Δ0, *his3*Δ1, *lys2*Δ0, *fox2::kanMX4* (Euroscarf, kindly provided by Daniel Brèthes, IBGC, Bordeaux). Media were prepared as described [26], [28]. Carbon assimilation tests were performed using YNB (0.17% (w/v) yeast nitrogen base without amino acids and without ammonium sulfate (Difco laboratories), 0.5% (w/v) ammonium sulfate) supplemented with 2% (w/v) of glucose (so called YNB-glucose) or of other carbon sources. Counterselection of uracil auxotrophs after pop-out of the *URA3* marker gene in transformants was done on YNB-glucose supplemented with 1 mg/ml of 5- fluoroorotic acid (5-FOA, Sigma Chemicals Co.) and 50 µg/ml uracil [26], [28]. For subcellular fractionation, we used the WOYglu medium, made of YNB supplemented with 0.1% (w/v) yeast extract and 0.3% (w/v) glucose, and the Induction medium, containing 0.5% (w/v) bactopeptone, 0.3% (w/v) yeast extract, 0.12% (v/v) oleic acid, 0.2% (v/v) Tween 40 and 0.5% (w/v) KH_2_PO_4_ (adjusted to pH 6.0 with NaOH), as described [54]. Yeasts were cultivated at 30°C, 35°C or 37°C, under constant agitation (230 rpm) for liquid cultures.

### PCR amplifications and gene cloning

*C. lusitaniae* genomic DNA was extracted as described [55] from spheroplasts prepared using zymolyase (Euromedex). Hot-Star (Qiagen) or Pfu (Promega) Taq DNA polymerase were used for the PCR amplification of DNA fragments. PCR were performed as recommended by the supplier. All the primers were synthesized by Eurofins MWG Operon and are listed in **S2 Table**. The complete *ICL1* and *FOX2* genes with their 5' and 3' non coding regions were isolated from the wild-type strain 6936 by PCR amplification and cloned into pGEM-T (Promega), to yield the plasmids pGICL1 and pGFOX2.

### *Icl1*Δ and *fox2*Δ mutant construction

*ICL1* and *FOX2* null mutants were constructed by using an integrative transformation system based upon the "*URA3*-blaster" strategy, adapted for *C. lusitaniae* [26]. The central part of the coding region of each cloned gene was deleted by digestion with adequate restriction enzymes from New England Biolabs (*Bcl*I and *Stu*I for *ICL1*, resulting in a 701-bp deletion; *Bgl*II and *Mfe*I for *FOX2*, resulting in a 1543-bp deletion) and was replaced by the GUN fragment; this fragment consisted of the *C. lusitaniae URA3* gene flanked on both sides by a noncoding 327-bp repeat (npt) obtained by amplification from the prokaryotic *NPTI* gene encoding neomycin phosphotransferase. The resulting disruption cassettes (*Icl1*Δ-GUN, *Fox2*Δ-GUN) were liberated from the plasmid backbone using *Nde*I and *Sph*I and were used separately to transform the strain *ura3*_[Δ360]_ to prototrophy, as previously described [26], in order to obtain the mutant strains *icl1*Δ*::*GUN and *fox2*Δ*::*GUN. All the genotypes were confirmed by PCR and Southern Blot hybridization, and for the *fox2*Δ locus, by nucleotide sequencing.

### Construction of the reintegrant strains *ICL1Re* and *FOX2Re*

Selection of mutant strains that had excised the *URA3* marker was achieved by plating yeast cells from the mutant strains *icl1*Δ::GUN and *fox2*Δ::GUN onto YNB glucose plates containing 5FOA and uracil. Uracil auxotrophic clones resistant to 5FOA were selected and their genetic organization, i.e. the loss of the *URA3* gene and of one of the flanking npt fragments, was confirmed by PCR and Southern Blot hybridization. The resulting mutant strains *icl1*Δ, *ura3*Δ and *fox2*Δ, *ura3*Δ were used further in two ways. First, they were transformed to uracil prototrophy by the complementation plasmid pG-URA3 to restore a functional *URA3* at its resident locus, allowing the selection of two new mutant strains having the genotypes *icl1*Δ, *ura3*_[Δ360]_::*URA3* (abbreviated *icl1*Δ) and *fox2*Δ, *ura3*_[Δ360]_::*URA3* (abbreviated *fox2*Δ). Second, the strains *icl1*Δ, *ura3*Δ and *fox2*Δ, *ura3*Δ were transformed to uracil prototrophy by the complementation plasmids pG-ICL1-URA3 and pG-FOX2-URA3, obtained after insertion of an *URA3* allele at the *Not*I and *Spe*I restriction site of the plasmids pG-ICL1 and pG-FOX2. Integration of the plasmids into the genome of the recipient strains was targeted at the *icl1*Δ and at the *fox2*Δ loci, respectively, to give the “reintegrant” strains *ICL1Re* and *FOX2Re*. All genotypes were verified by PCR and Southern Blot hybridization; the genotype of the *fox2*Δ strain was also controlled by nucleotide sequencing of the deleted locus.

### Disruption of the *PXA1* gene of *C. lusitaniae*

The *pxa1*Δ mutant was constructed by disruption of the coding sequence of the wild allele. To do that, a 564-bp fragment corresponding to the central part of the coding region of *PXA1* was amplified by PCR and cloned to the *Nco*I and *Sac*II restriction sites of the plasmid pG-URA3 (corresponding to a pGEM-T plasmid harbouring the *C. lusitaniae URA3* gene). The resulting plasmid pG-URA3-pxa1_[core]_ was then linearized by *Afl*II, a single restriction site localized within the *PXA1* cloned sequence, before transforming the strain *ura3*_[Δ990]_ to prototrophy. The *pxa1*Δ::*URA3* mutant strain was obtained after homologous integration of the plasmid at the *PXA1* locus. *PXA1* was deleted in a wild type and in a *fox2*Δ genetic background.

### Southern hybridizations

At least 2 µg of *C. lusitaniae* DNA was digested with *Hind*III or *Eco*RV and separated by electrophoresis in a 0.8% agarose gel. The DNA was transferred onto a nylon membrane (Hybond N+; Roche Molecular Biochemicals) and hybridized with digoxigenin-labeled DNA probes synthesized with a PCR DIG probe synthesis kit (Roche Molecular Biochemicals), as recommended by the supplier. *ICL1*, *FOX2*, *PXA1* and *URA3* DNA probes were generated by PCR amplification with the relevant primers (**S2 Table**). The *bla* DNA probe, which was specific to the plasmidic ampicillin resistance gene, was also generated with specific primers, as described previously [26].

### DNA sequencing and sequence analysis

Sequence reaction was realized using ABI Prism Dye Terminator Cycle Sequencing Ready Reaction v1.1 Kit (Applied Biosystems) from plasmidic DNA (300 à 600 ng) or PCR amplification (100 ng), as recommended by the supplier. Sequencing was done by the genotyping-sequencing facility of the Bordeaux University. The similarity searches in databases were performed with the Basic Local Alignment Search Tool (BLAST) programs [36], available on the websites of the National Center for Biotechnology Information (http://www.ncbi.nlm.nih.gov) and of the BROAD Institute (http://www.broadinstitute.org/annotation/genome/candida_group/Blast.html). Consensus multiple alignments for nucleotide and amino acid sequences was performed with the ClustalW program [56], available on the European Bioinformatics Institute website (http://www.ebi.ac.uk/Tools/clustalw2/index.html). Genbank nucleotide sequence accession numbers are JQ710936 for *ICL1*, JQ710937 for *FOX2* and JQ710938 for *PXA1*.

### *In vitro* growth assays

Growth assays were performed on solid YNB media containing 2% of either glucose, potassium acetate, ethanol, citrate, glycerol, oleic acid (C18:1), stearic acid (C18:0), palmitic acid (C16:0), myristic acid (C14:0), lauric acid (C12:0), or capric acid (C10:0) as the sole carbon source. Cultures were incubated at 30°C for 3 to 7 days, depending on the carbon source, as indicated in the figure legends. For the drop tests, the strains were grown to mid-log phase in YPD, collected by centrifugation, washed with water, and resuspended in water to an OD_600_ of 0.5. Cells were transferred to 96-well plates, and serially diluted fivefold (1∶5). Drops of 5 µl of each dilution were deposited onto solid media. For growth assays in liquid media, strains were grown in YNB-glucose at 30°C overnight. The next day, cells were harvested by centrifugation, washed twice with water, and resuspended at an OD_600_ of 0.08 in YNB media containing the appropriate carbon source. Growth of the cultures was assessed over a period of 5 to 7 days by measuring the OD at 600 nm.

### Lipid analysis

After growth on YPD or YNB +2% palmitic acid, yeast were harvested and washed 3 times in water; amount of cells equivalent to 15 mg dry weight were then converted to spheroplasts using zymolyase, as described above. To extract lipids from whole cells, two ml of chloroform/methanol (2∶1, v/v) were added to the cell suspensions. After shaking and centrifugation, the organic phase was removed and the lipids contained in the remaining aqueous phase were further extracted twice by the addition of 2 ml of chloroform. The organic phases were then pooled and evaporated to dryness. The lipids were then dissolved in 70 µL of chloroform / methanol (2∶1 v/v). Neutral lipids were separated and analyzed by one-dimensional thin-layer chromatography (TLC) on silica gel plates (10×10 cm; Merck) using hexane / diethylether / acetic acid (90: 15: 2) as solvent. The lipids were then visualized by spraying the plates with a solution of 0.001% (w/v) primuline in 80% acetone, followed by exposure of the plates to UV light. The silica gel zones corresponding to the various lipids were then scraped from the plates and added to 1 mL of methanol / 2.5% H_2_SO_4_ containing 5 µg of heptadecanoic acid. After 1 h at 80°C, 1.5 mL of H_2_O was added and fatty acid methyl esters (FAMES) were extracted with 0.75 mL of hexane. Separation of FAMES was performed by gas chromatography (GC) (Hewlett Packard 5890 series II; Hewlett-Packard, Palo Alto, CA, USA).

### Purification of organelle fractions with sucrose density gradient

Preparation of subcellular fraction was adapted from Distel & Kragt protocol [54], except that **i**/the conversion of yeast cells to spheroplasts was done in Digestion Buffer (Sorbitol 1.35 M, EDTA 1 mM, Citrate phosphate 10 mM, pH 5.8) supplemented with 10 mg zymolyase per gram of fresh cells and that **ii**/the spheroplasts were broken by incubation 1 h on ice in 10 ml of homogenization buffer (Sorbitol 0.6 M in KEM (KCl 1 mM, EDTA 1 mM, MES 5 mM), PMSF 1 mM) per gram of initial fresh cells. Sucrose density gradients were adapted from Kamiryo *et al*. [57]. The organelle fraction was applied onto a discontinuous gradient consisting of 4.5 ml, 4.5 ml, 9 ml, and 4.5 ml of sucrose solutions at 25, 35, 42, and 53% (w/w in KEM), respectively. The tubes were centrifuged at 100,000 g_av_ for 1 h with a AH-629 swinging-bucket rotor (Sorvall). The band at the 53 to 42% sucrose interphase corresponded to the peroxisomal fraction, and the band at the 42 to 35% interphase contained the mitochondrial fraction. Protein concentrations were estimated by the Bradford method [58], using bovine serum albumin as standard.

### Enzymatic assays

Catalase (EC 1.11.1.6) activity was assayed using a Cary 100 scan (Varian) spectrophotometer at λ = 240 nm and at 20°C, as described by Aebi [59]. The reaction mixture contained 10 µg protein sample, 50 mM potassium phosphate (pH 7) and 11 mM H_2_0_2_ was used to start the reaction. Cytochrome c oxidase (EC 1.9.3.1) activity was measured at λ = 550 nm and 37°C, following a method adapted from Polakis *et al*. [60]. Cytochrome *c* (Sigma) was previously reduced by equimolar addition of ascorbic acid. The reaction mixture contained 100 mM potassium phosphate (pH 6.9), 1 mM EDTA, and 28 µM reduced cytochrome *c*. The reaction was started with the addition of the protein sample. One enzyme unit is defined as the amount which catalyzes the conversion of 1 µmol of substrate per min.

### Expression and purification of *C. lusitaniae* Fox2p in *E. coli*

The coding region of *FOX2* was amplified by PCR with the primers FpET28NdeFox2 and RpET28XhoFox2. The 2747 bp amplicon and the pET-28a(+) plasmid (Novagen, Merck4biosciences, Germany) were then digested with *Nde*I and *Xho*I, ligated and cloned in *E. coli* Stellar HST04 (Clontech Laboratories Inc.). The recombinant vector was verified by nucleotide sequencing, and cloned in *E. coli* BL21 Star (Novagen). Expression of the gene encoding the histidine-tagged Fox2p protein was induced using 0.5 mM isopropyl-β-D-thiogalactopyranoside at 22°C during 16 h. Bacterial cells were harvested by centrifugation (5,000 g, 10 min). Typically, a bacterial pellet obtained from 1 liter of culture was disrupted by sonication in 15 ml of extraction buffer (Na_2_HPO_4_ 20 mM pH 7.6, 10% (v/v) glycerol, 0.3 M NaCl, 1 mM β-mercaptoethanol, 0.2% (v/v) Triton-X100). The cellular lysate was centrifuged (5,000 g, 5 min). The His-tagged Fox2p was purified by immobilized-metal affinity chromatography: the supernatant was loaded on a Ni Sepharose High Performance HiTrap column (GE Healthcare), previously equilibrated in buffer A (Na_2_HPO_4_ 50 mM, NaCl 0.3 M, pH 7.6). After washing with 5 column volumes of buffer A, the retained protein was eluted in buffer A with increasing concentrations of imidazole from 0 to 0.25 M. The pool of fractions containing His-tagged Fox2p was dialyzed against PBS at 4°C during 16 h, reloaded on the HiTrap column and incubated with thrombin (10 units/µg of protein) during 16 h at room temperature. The detagged Fox2p was then eluted with buffer A.

### Production and purification of hen IgY polyclonal antibodies anti-*C. lusitaniae* Fox2p

The immunization of two hens (Eurogentec, Seraing, Belgium) was performed with 4 successive injections 15 days apart of 100 µg of purified Fox2p. Three egg yolk collections were obtained from Day 38 to 52, Day 76 to 90 and Day 91 to 105 after the first injection. IgY were then purified from egg yolk by precipitation in the presence of increasing concentrations of polyethylene glycol (PEG 6000) according to Pauly *et al*. [61]. After a first dialysis under gentle agitation at 4°C against 0.1% (w/v) NaCl during 16 h, and a second dialysis against PBS during 1 h, purified antibodies were stored at −20°C in 50% (w/v) glycerol, 0.1% (w/v) BSA and 0.01% (w/v) azide.

### Two-step affinity purification of polyclonal hen anti-Fox2p IgY

**i**/Purified Fox2p was spotted on a polyvinylidene difluoride (PVDF) membrane (BioRad). After room temperature drying, the membrane was saturated in PBS containing 0.05% (v/v) Tween 40 and 5% (w/v) dehydrated milk during 1 h and incubated 16 h at 4°C with anti-Fox2p IgY. The membrane was washed 3 times (10 min) in 1 M NaCl. Purified antibodies were eluted with 200 mM glycine pH 2.5, extemporaneously buffered with 2 M unPhed-Tris (Tris-glycine ratio 1∶10), and dialyzed as mentioned above. **ii**/Mitochondrial protein fractions of *fox2*Δ cells were deposited on a PVDF membrane. Membrane was dryed, saturated as described above and incubated 16 h at 4°C with antibodies previously purified against Fox2p. The solution of antibodies was then collected, dialyzed and stored as mentioned above.

### Other primary antibodies used in this study

Polyclonal rabbit antiserums were used in this study: **i**/against Fox2p [62], designed against the *C. tropicalis* [17] multifunctional beta-oxidation enzyme, and kindly provided by W. Schliebs (Medizinisch Fakultät Ruhr- Universität Bochum, Germany), **ii**/against Icl1p [63], designed against the *Ashbya gossypii* isocitrate lyase, and kindly provided by S. Nieland (Hochschule Lausitz, Senftenberg, Germany), **iii**/against Cyt*c*, designed against the *S. cerevisiae* Cyt*c* [64].

### Immunodetection of Fox2p and Icl1p by western-blotting

Ten µg of proteins of peroxisomal and mitochondrial fractions or 35 µg of proteins of crude cellular extracts were separated by SDS-PAGE (sodium dodecyl sulfate - polyacrylamide gel electrophoresis), as described by Laemmli [65], and transferred to PVDF membrane. Polyclonal primary rabbit antiserums against *C. tropicalis* Fox2p and against *Ashbya gossypii* Icl1p were used. The immunodetection was realized after incubation with a secondary anti-rabbit antibody coupled with peroxidase (Sigma-Aldrich) using Clarity Western ECL Substrate (BioRad) and ImageQuant LAS4000 (GE Healthcare). The ImageQuant TL software (GE Healthcare) was used for the signal quantification.

### Palmitoyl-CoA catabolism assay

Crude cellular extracts (35 µg) or organelle fraction lysates (10 µg) were incubated at 37°C in 100 µL reaction mixture containing 20 mM NAD, 10 mM CoA-SH, 1 mM FAD, 100 mM KCN, 2% Triton, Tris-HCl 100 mM pH 7.4. The reaction was started by addition of 1 µL of ^14^C_α_-palmitoyl-CoA 10 µM (20 µCi/ml, Sigma-Aldrich). The reaction was stopped by addition of 100 µL KOH 5 M. After saponification (one hour at 65°C), and acidification (addition of 100 µL H_2_SO_4_ 36 N), FA were extracted with 2 mL of CHCl_3_. Extracts were evaporated (under N_2_), dissolved in 50 µL CHCl_3_/CH_3_OH 2∶1 (v/v) and the compounds were separated by thin layer chromatography (TLC) on 10×10 cm silica gel plates (Merck) in 75∶24∶1 hexane/diethyl ether/acetic acid (v/v/v). Revelation was done using Phosphorimager SI system (Amersham Biosciences). Analysis of ^14^C_α_-palmitoyl-CoA consumption were done using ImageQuant analysis software (Amersham Biosciences).

### Electron microscopy

After growth on YPD or induction medium according to the protocol used for peroxisome purification [54], cells were harvested and placed on the surface of Formvar-coated copper grids (HS400 Pelanne instruments 400 mesh). The grids were then quickly submersed in liquid propane (−180°C). Sample preparation for electron microscopy and immunoelectron microscopy were performed as previously described [66]. Samples were observed with a Philips Tecnai 12 Biotwin (120 kV) electron microscope at the Bordeaux Imaging Center.

### Statistical analysis

All the data obtained were analyzed using the two-tailed Student's t-test with a statistical significance at p≤0.05. All assays and experiments were carried out at least in triplicate.

## Supporting Information

S1 Figure **Southern blot analysis of the wild-type,** ***fox2*** **Δ and** ***FOX2*** **reintegrant strains.** (A) Panel showing the genetic maps of the *FOX2* (left panel) and *URA3* (right panel) loci of three mutant strains (*fox2*Δ::GUN, *fox2*Δ *ura3*Δ, *fox2*Δ), of the reintegrant strain (*FOX2Re*), and of the wild-type strain (6936). Sequences homologous to *FOX2* are colored in black, npt sequences (noncoding 327-bp fragments derived from the prokaryotic *NPT1* gene encoding neomycin phosphotransferase) are shown in hatched box, sequences homologous to *URA3* are colored in grey, pGEM-T sequence is colored in white. Genomic DNA was digested with *EcoR*V (arrows) and analyzed by Southern blotting using: (B) the *FOX2* probe (homologous to the entire sequence of *FOX2*) and (C) *URA3* probe (homologous to the entire sequence of *URA3*).
(PDF)

S2 Figure **Growth tests of wild-type and mutant strains of** ***C. lusitaniae*** **on YNB agar supplemented or not with different carbon sources.**
(PDF)

S3 Figure **Growth of** ***C. lusitaniae*** **and** ***S. cerevisiae*** **wild type and mutant strains in liquid YNB + 2% (v/v) C18:1.** OD: optical density, WT: wild-type. For growth of the *S. cerevisiae* Y15080 *fox2* strain, the medium was supplemented with 25 µg/ml of lysine, uracil, leucine and histidine.
(PDF)

S1 Table **Genotypes of the** ***Candida lusitaniae*** **strains used in this study.**
(PDF)

S2 Table **Oligonucleotides used in this study.**
(PDF)

S1 File **Identification of the** ***ICL1*** **,** ***FOX2*** **and** ***PXA1*** **genes in the genome of** ***C. lusitaniae*** **.**
(DOCX)

## References

[1] Singh A, Del Poeta M (2011) Lipid signalling in pathogenic fungi. Cell Microbiol 13:177–185. 21091925 10.1111/j.1462-5822.2010.01550.x PMC5142819

[2] Brock M (2009) Fungal metabolism in host niches. Curr Opin Microbiol 12:371–376. 19535285 10.1016/j.mib.2009.05.004

[3] Strijbis K, Distel B (2010) Intracellular acetyl unit transport in fungal carbon metabolism. Eukaryot Cell 9:1809–1815. 20889721 10.1128/EC.00172-10 PMC3008284

[4] Hiltunen JK, Mursula AM, Rottensteiner H, Wierenga RK, Kastaniotis AJ, et al (2003) The biochemistry of peroxisomal beta-oxidation in the yeast *Saccharomyces cerevisiae* . FEMS Microbiol Rev 27:35–64. 12697341 10.1016/S0168-6445(03)00017-2

[5] Moreno de la Garza M, Schultz-Borchard U, Crabb JW, Kunau W-H (1985) Peroxisomal beta-oxidation system of *Candida tropicalis* . Eur J Biochem 148:285–291. 3987689 10.1111/j.1432-1033.1985.tb08837.x

[6] Kurihara T, Ueda M, Okada H, Kamasawa N, Naito N, et al (1992) Beta-oxidation of butyrate, the short-chain-length fatty acid, occurs in peroxisomes in the yeast *Candida tropicalis* . J Biochem 111:783–787. 1500419 10.1093/oxfordjournals.jbchem.a123836

[7] Smith JJ, Brown TW, Eitzen GA, Rachubinski RA (2000) Regulation of peroxisome size and number by fatty acid beta-oxidation in the yeast *Yarrowia lipolytica* . J Biol Chem 275:20168–20178. 10787422 10.1074/jbc.M909285199

[8] Kunau W-H, Dommes V, Schulz H (1995) Beta-oxidation of fatty acids in mitochondria, peroxisomes, and bacteria: A century of continued progress. Prog Lipid Res 34:267–342. 8685242 10.1016/0163-7827(95)00011-9

[9] Shen Y-Q, Burger G (2009) Plasticity of a key metabolic pathway in fungi. Funct Integr Genomics 9:145–151. 18795352 10.1007/s10142-008-0095-6

[10] Hettema EH, Van Roermund CWT, Distel B, Van Den Berg M, Vilela C, et al (1996) The ABC transporter proteins Pat1 and Pat2 are required for import of long-chain fatty acids into peroxisomes of *Saccharomyces cerevisiae* . EMBO J 15:3813–3822. 8670886 PMC452064

[11] Shani N, Watkins PA, Valle D (1995) *PXA1*, a possible *Saccharomyces cerevisiae* ortholog of the human adrenoleukodystrophy gene. Proc Natl Acad Sci U S A 92:6012–6016. 7597071 10.1073/pnas.92.13.6012 PMC41632

[12] Hettema EH, Tabak HF (2000) Transport of fatty acids and metabolites across the peroxisomal membrane. Biochim Biophys Acta 1486:18–27. 10856710 10.1016/s1388-1981(00)00045-7

[13] Cohen G, Fessl F, Traczyk A, Rytka J, Ruis H (1985) Isolation of the catalase A gene of *Saccharomyces cerevisiae* by complementation of the cta1 mutation. Mol Gen Genet 200:74–79. 3897793 10.1007/BF00383315

[14] Hiltunen JK, Wenzel B, Beyer A, Erdmann R, Fosså A, et al (1992) Peroxisomal multifunctional beta-oxidation protein of *Saccharomyces cerevisiae*. Molecular analysis of the fox2 gene and gene product. J Biol Chem 267:6646–6653. 1551874

[15] Ylianttila MS, Pursiainen NV, Haapalainen AM, Juffer AH, Poirier Y, et al (2006) Crystal structure of yeast peroxisomal multifunctional enzyme: structural basis for substrate specificity of (3R)-hydroxyacyl-CoA dehydrogenase units. J Mol Biol 358:1286–1295. 16574148 10.1016/j.jmb.2006.03.001

[16] Lorenz MC, Fink GR (2001) The glyoxylate cycle is required for fungal virulence. Nature 412:83–86. 11452311 10.1038/35083594

[17] Thieringer R, Kunau WH (1991) Beta-oxidation system of the filamentous fungus *Neurospora crassa*. Structural characterization of the trifunctional protein. J Biol Chem 266:13118–13123. 1830049

[18] Baltazar MF, Dickinson FM, Ratledge C (1999) Oxidation of medium-chain acyl-CoA esters by extracts of *Aspergillus niger*: enzymology and characterization of intermediates by HPLC. Microbiol 145:271–278. 10.1099/13500872-145-1-271 10206707

[19] Maggio-Hall LA, Keller NP (2004) Mitochondrial beta-oxidation in *Aspergillus nidulans* . Mol Microbiol 54:1173–1185. 15554960 10.1111/j.1365-2958.2004.04340.x

[20] Boisnard S, Espagne E, Zickler D, Bourdais A, Riquet A-L, et al (2009) Peroxisomal ABC transporters and beta-oxidation during the life cycle of the filamentous fungus *Podospora anserina* . Fungal Genet Biol 46:55–66. 18992353 10.1016/j.fgb.2008.10.006

[21] Feron G, Blin-Perrin C, Krasniewski I, Mauvais G, Lherminier J (2005) Metabolism of fatty acid in yeast: characterisation of beta-oxidation and ultrastructural changes in the genus *Sporidiobolus* sp. cultivated on ricinoleic acid methyl ester. FEMS Microbiol Lett 250:63–69. 16043312 10.1016/j.femsle.2005.06.045

[22] Cornell MJ, Alam I, Soanes DM, Wong HM, Hedeler C, et al (2007) Comparative genome analysis across a kingdom of eukaryotic organisms: Specialization and diversification in the Fungi. Genome Res 17:1809–1822. 17984228 10.1101/gr.6531807 PMC2099590

[23] Favel A, Michel-Nguyen A, Peyron F, Martin C, Thomachot L, et al (2003) Colony morphology switching of *Candida lusitaniae* and acquisition of multidrug resistance during treatment of a renal infection in a newborn: case report and review of the literature. Diagn Microbiol Infect Dis 47:331–339. 12967746 10.1016/s0732-8893(03)00094-4

[24] Rodrigues de Miranda L (1979) *Clavispora*, a new yeast genus of the *Saccharomycetales* . Antonie van Leeuwenhoek 45:479–483. 554535 10.1007/BF00443285

[25] François F, Noël T, Pépin R, Brulfert A, Chastin C, et al (2001) Alternative identification test relying upon sexual reproductive abilities of *Candida lusitaniae* strains isolated from hospitalized patients. J Clin Microbiol 39:3906–3914. 11682506 10.1128/JCM.39.11.3906-3914.2001 PMC88463

[26] François F, Chapeland-Leclerc F, Villard J, Noël T (2004) Development of an integrative transformation system for the opportunistic pathogenic yeast *Candida lusitaniae* using *URA3* as a selection marker. Yeast 21:95–106. 14755635 10.1002/yea.1059

[27] Shen J, Guo W, Kohler JR (2005) *CaNAT1*, a heterologous dominant selectable marker for transformation of *Candida albicans* and other pathogenic *Candida* species. Infect Immun 73:1239–1242. 15664973 10.1128/IAI.73.2.1239-1242.2005 PMC547112

[28] El-Kirat-Chatel S, Dementhon K, Noël T (2011) A two-step cloning-free PCR-based method for the deletion of genes in the opportunistic pathogenic yeast *Candida lusitaniae* . Yeast 28:321–30. 21456057 10.1002/yea.1836

[29] Roetzer A, Gratz N, Kovarik P, Schüller C (2010) Autophagy supports *Candida glabrata* survival during phagocytosis. Cell Microbiol 12:199–216. 19811500 10.1111/j.1462-5822.2009.01391.x PMC2816358

[30] Dementhon K, El-Kirat-Chatel S, Noël T (2012) Development of an in vitro model for the multi-parametric quantification of the cellular interactions between *Candida* yeasts and phagocytes. PLoS ONE 7:e32621. 22479332 10.1371/journal.pone.0032621 PMC3316538

[31] Prigneau O, Porta A, Poudrier JA, Colonna-Romano S, Noël T, et al (2003) Genes involved in beta-oxidation, energy metabolism and glyoxylate cycle are induced by *Candida albicans* during macrophage infection. Yeast 20:723–730. 12794933 10.1002/yea.998

[32] Lorenz MC, Bender JA, Fink GR (2004) Transcriptional response of *Candida albicans* upon internalization by macrophages. Eukaryot Cell 3:1076–1087. 15470236 10.1128/EC.3.5.1076-1087.2004 PMC522606

[33] Wang Z-Y, Thornton CR, Kershaw MJ, Debao L, Talbot NJ (2003) The glyoxylate cycle is required for temporal regulation of virulence by the plant pathogenic fungus *Magnaporthe grisea* . Mol Microbiol 47:1601–1612. 12622815 10.1046/j.1365-2958.2003.03412.x

[34] Piekarska K, Mol E, van den Berg M, Hardy G, van den Burg J, et al (2006) Peroxisomal fatty acid beta-oxidation is not essential for virulence of *Candida albicans* . Eukaryot Cell 5:1847–1856. 16963628 10.1128/EC.00093-06 PMC1694795

[35] Ramírez MA, Lorenz MC (2007) Mutations in alternative carbon utilization pathways in *Candida albicans* attenuate virulence and confer pleiotropic phenotypes. Eukaryot Cell 6:280–290. 17158734 10.1128/EC.00372-06 PMC1797957

[36] Altschul S, Madden T, Schaffer A, Zhang J, Zhang Z, et al (1997) Gapped BLAST and PSI-BLAST: a new generation of protein database search programs. Nucl Acids Res 25:3389–3402. 9254694 10.1093/nar/25.17.3389 PMC146917

[37] Beopoulos A, Cescut J, Haddouche R, Uribelarrea J-L, Molina-Jouve C, et al (2009) *Yarrowia lipolytica* as a model for bio-oil production. Prog Lipid Res 48:375–387. 19720081 10.1016/j.plipres.2009.08.005

[38] Gurvitz A, Rottensteiner H (2006) The biochemistry of oleate induction: Transcriptional upregulation and peroxisome proliferation. Biochim Biophys Acta 1763:1392–1402. 16949166 10.1016/j.bbamcr.2006.07.011

[39] Piekarska K, Hardy G, Mol E, van den Burg J, Strijbis K, et al (2008) The activity of the glyoxylate cycle in peroxisomes of *Candida albicans* depends on a functional beta-oxidation pathway: evidence for reduced metabolite transport across the peroxisomal membrane. Microbiol 154:3061–3072. 10.1099/mic.0.2008/020289-0 18832312

[40] Dommes P, Dommes V, Kunau WH (1983) beta-Oxidation in *Candida tropicalis*. Partial purification and biological function of an inducible 2,4-dienoyl coenzyme A reductase. J Biol Chem 258:10846–10852. 6885804

[41] Maggio-Hall LA, Lyne P, Wolff JA, Keller NP (2008) A single acyl-CoA dehydrogenase is required for catabolism of isoleucine, valine and short-chain fatty acids in *Aspergillus nidulans* . Fungal Genet Biol 45:180–189. 17656140 10.1016/j.fgb.2007.06.004 PMC2905684

[42] Eaton S, Bursby T, Middleton B, Pourfarzam M, Mills K, et al (2000) The mitochondrial trifunctional protein: centre of a beta-oxidation metabolon? Biochem Soc Trans 28:177–182. 10816122 10.1042/bst0280177

[43] Neuberger G, Maurer-Stroh S, Eisenhaber B, Hartig A, Eisenhaber F (2003) Prediction of peroxisomal targeting signal 1 containing proteins from amino acid sequence. J Mol Biol 328:581–592. 12706718 10.1016/s0022-2836(03)00319-x

[44] Ast J, Stiebler AC, Freitag J, Bölker M (2013) Dual targeting of peroxisomal proteins. Front Physiol 4:297. 24151469 10.3389/fphys.2013.00297 PMC3798809

[45] Yogev O, Pines O (2011) Dual targeting of mitochondrial proteins: Mechanism, regulation and function. Biochim Biophys Acta 1808:1012–1020. 20637721 10.1016/j.bbamem.2010.07.004

[46] Ferdinandusse S, Denis S, van Roermund CWT, Wanders RJA, Dacremont G (2004) Identification of the peroxisomal beta-oxidation enzymes involved in the degradation of long-chain dicarboxylic acids. J Lipid Res 45:1104–1111. 15060085 10.1194/jlr.M300512-JLR200

[47] Cheng Q, Sanglard D, Vanhanen S, Liu HT, Bombelli P, et al (2005) *Candida* yeast long chain fatty alcohol oxidase is a c-type haemoprotein and plays an important role in long chain fatty acid metabolism. Biochim Biophys Acta 1735:192–203. 16046182 10.1016/j.bbalip.2005.06.006

[48] Picataggio S, Rohrer T, Deanda K, Lanning D, Reynolds R, et al (1992) Metabolic engineering of *Candida tropicalis* for the production of long-chain dicarboxylic acids. Biotechnology (N Y) 10:894–898. 1368984 10.1038/nbt0892-894

[49] Foulon V, Antonenkov VD, Croes K, Waelkens E, Mannaerts GP, et al (1999) Purification, molecular cloning, and expression of 2-hydroxyphytanoyl-CoA lyase, a peroxisomal thiamine pyrophosphate-dependent enzyme that catalyzes the carbon–carbon bond cleavage during α-oxidation of 3-methyl-branched fatty acids. Proc Natl Acad Sci USA 96:10039–10044. 10468558 10.1073/pnas.96.18.10039 PMC17838

[50] Jansen GA, Wanders RJA (2006) Alpha–Oxidation. Biochim Biophys Acta 1763:1403–1412. 16934890 10.1016/j.bbamcr.2006.07.012

[51] Wanders RJ, van Roermund CW, Jakobs C, ten Brink HJ (1991) Identification of pristanoyl-CoA oxidase and phytanic acid decarboxylation in peroxisomes and mitochondria from human liver: implications for Zellweger syndrome. J Inherit Metab Dis 14:349–352. 1770789 10.1007/BF01811700

[52] Refsum S (1947) Heredopathia atactica polyneuritiformis: A familial syndrome not hitherto described. A contribution to the clinical study of the hereditary diseases of the nervous system. J Am Med Assoc 133:1319 doi:10.1001/jama.1947.02880170065026.

[53] Shen Y-Q, Lang BF, Burger G (2009) Diversity and dispersal of a ubiquitous protein family: acyl-CoA dehydrogenases. Nucleic Acids Res 37:5619–5631. 19625492 10.1093/nar/gkp566 PMC2761260

[54] Distel B, Kragt A (2006) Purification of yeast peroxisomes. Methods Mol Biol 313:21–26. 16118420 10.1385/1-59259-958-3:021

[55] Scherer S, Stevens DA (1987) Application of DNA typing methods to epidemiology and taxonomy of *Candida* species. J Clin Microbiol 25:675–679. 3033016 10.1128/jcm.25.4.675-679.1987 PMC266058

[56] Thompson JD, Higgins DG, Gibson TJ (1994) CLUSTAL W: improving the sensitivity of progressive multiple sequence alignment through sequence weighting, position-specific gap penalties and weight matrix choice. Nucl Acids Res 22:4673–4680. 7984417 10.1093/nar/22.22.4673 PMC308517

[57] Kamiryo T, Abe M, Okazaki K, Kato S, Shimamoto N (1982) Absence of DNA in peroxisomes of *Candida tropicalis* . J Bacteriol 152:269–274. 7118828 10.1128/jb.152.1.269-274.1982 PMC221401

[58] Bradford MM (1976) A rapid and sensitive method for the quantitation of microgram quantities of protein utilizing the principle of protein-dye binding. Anal Biochem 72:248–254. 942051 10.1016/0003-2697(76)90527-3

[59] Aebi H (1984) Catalase in vitro. Meth Enzymol 105:121–126. 6727660 10.1016/s0076-6879(84)05016-3

[60] Polakis ES, Bartley W, Meek GA (1964) Changes in the structure and enzyme activity of *Saccharomyces cerevisiae* in response to changes in the environment. Biochem J 90:369–374. 4284219 10.1042/bj0900369 PMC1202626

[61] Pauly D, Chacana PA, Calzado EG, Brembs B, Schade R (2011) IgY technology: Extraction of chicken antibodies from egg yolk by polyethylene glycol (PEG) precipitation. J Vis Exp 51 pii: 3084. doi: 10.3791/3084. 10.3791/3084 PMC3197133 21559009

[62] Fosså A, Beyer A, Pfitzner E, Wenzel B, Kunau WH (1995) Molecular cloning, sequencing and sequence analysis of the fox-2 gene of *Neurospora crassa* encoding the multifunctional beta-oxidation protein. Mol Gen Genet 247:95–104. 7715608 10.1007/BF00425825

[63] Schmidt G, Stahmann K-P, Kaesler B, Sahm H (1996) Correlation of isocitrate lyase activity and riboflavin formation in the riboflavin overproducer *Ashbya gossypii* . Microbiol 142:419–426. 10.1099/13500872-142-2-419 33657747

[64] Pereira C, Chaves S, Alves S, Salin B, Camougrand N, et al (2010) Mitochondrial degradation in acetic acid-induced yeast apoptosis: the role of Pep4 and the ADP/ATP carrier. Mol Microbiol 76:1398–1410. 20345665 10.1111/j.1365-2958.2010.07122.x

[65] Laemmli UK (1970) Cleavage of structural proteins during the assembly of the head of bacteriophage T4. Nature 227:680–685. 5432063 10.1038/227680a0

[66] Camougrand N, Kiššová I, Salin B, Devenish RJ (2008) Monitoring mitophagy in yeast. In:Daniel J Klionskyeditor. Methods in Enzymology. Autophagy: Lower Eukaryotes and Non-Mammalian Systems, Part A. Academic Press, Vol. Volume 451 pp. 89–107. 10.1016/S0076-6879(08)03208-4 19185716

