# Supplementary material for: A Fox2-Dependent Fatty Acid ß-Oxidation Pathway Coexists Both in Peroxisomes and Mitochondria of the Ascomycete Yeast Candida lusitaniae
Source: PLoS One. 2014 Dec 8;9(12):e114531. doi: 10.1371/journal.pone.0114531 (PMC4259357; doi:10.1371/journal.pone.0114531)
Supplement: S1 Figure — Southern blot analysis of the wild-type, fox2 Δ and FOX2 reintegrant strains. (A) Panel showing the genetic maps of the FOX2 (left panel) and URA3 (right panel) loci of three mutant strains (fox2Δ::GUN, fox2Δ ura3Δ, fox2Δ), of the reintegrant strain (FOX2Re), and of the wild-type strain (6936). Sequences homologous to FOX2 are colored in black, npt sequences (noncoding 327-bp fragments derived from the prokaryotic NPT1 gene encoding neomycin phosphotransferase) are shown in hatched box, sequences homologous to URA3 are colored in grey, pGEM-T sequence is colored in white. Genomic DNA was digested with EcoRV (arrows) and analyzed by Southern blotting using: (B) the FOX2 probe (homologous to the entire sequence of FOX2) and (C) URA3 probe (homologous to the entire sequence of URA3). (PDF) [file pone.0114531.s001.pdf]

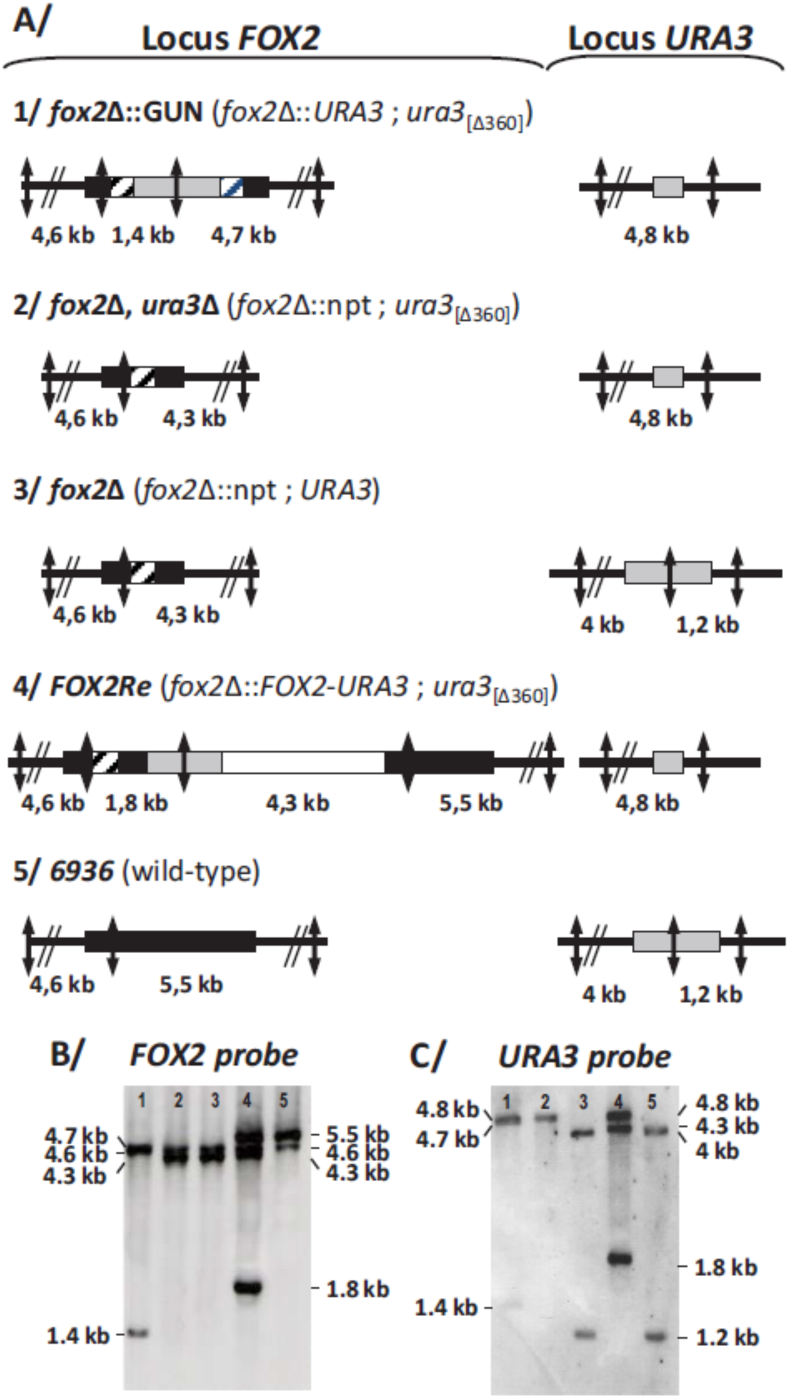

A/
Locus FOX2
Locus URA3
1/ fox2Δ::GUN (fox2Δ::URA3 ; ura3[Δ360])
4,6 kb 1,4 kb 4,7 kb
4,8 kb
2/ fox2Δ, ura3Δ (fox2Δ::npt ; ura3[Δ360])
4,6 kb 4,3 kb
4,8 kb
3/ fox2Δ (fox2Δ::npt ; URA3)
4,6 kb 4,3 kb
4 kb 1,2 kb
4/ FOX2Re (fox2Δ::FOX2-URA3 ; ura3[Δ360])
4,6 kb 1,8 kb 4,3 kb 5,5 kb
4,8 kb
5/ 6936 (wild-type)
4,6 kb 5,5 kb
4 kb 1,2 kb
B/ FOX2 probe
1 2 3 4 5
4.7 kb
4.6 kb
4.3 kb
1.4 kb
5.5 kb
4.6 kb
4.3 kb
1.8 kb
C/ URA3 probe
1 2 3 4 5
4.8 kb
4.7 kb
1.4 kb
4.8 kb
4.3 kb
4 kb
1.8 kb
1.2 kb

Figure S1.

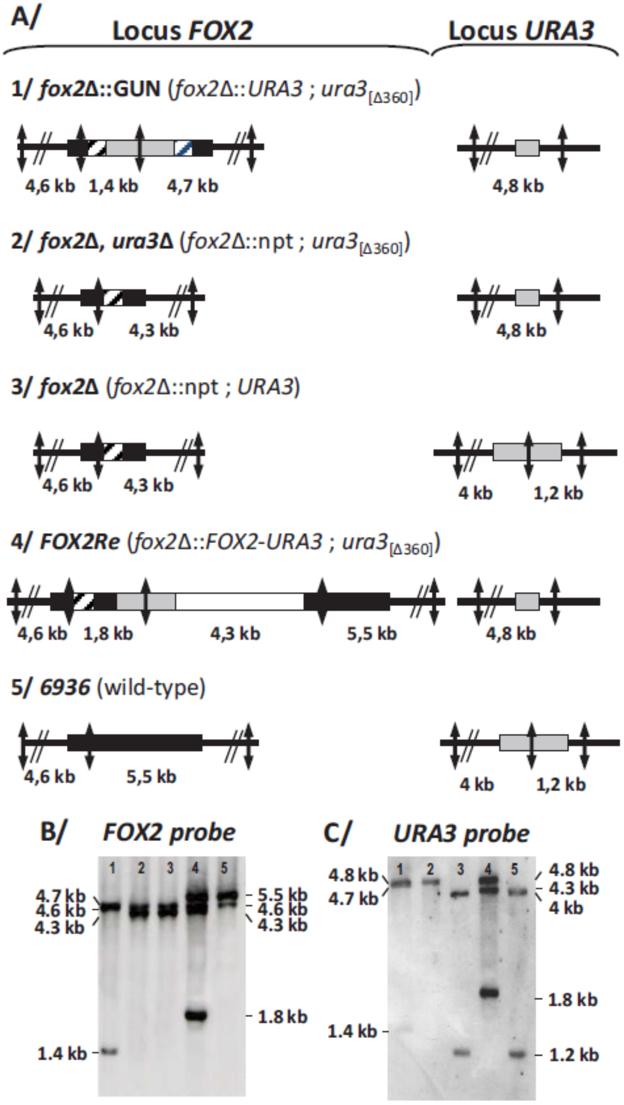

Fig. S1. Southern blot analysis of the wild-type and *fox2*Δ strains. (A) Panel showing the genetic maps of the *FOX2* (left panel) and *URA3* (right panel) loci of three mutant strains (*fox2*Δ::GUN, *fox2*Δ *ura3*Δ, *fox2*Δ), of the reintegrant strain (*FOX2Re*), and of the wild-type strain (6936). Sequences homologous to *FOX2* are colored in black, npt sequences (noncoding 327-bp fragments derived from the prokaryotic *NPT1* gene encoding neomycin phosphotransferase) are shown in hatched box, sequences homologous to *URA3* are colored in grey,
