# Supplementary figures and images for: A Fox2-Dependent Fatty Acid ß-Oxidation Pathway Coexists Both in Peroxisomes and Mitochondria of the Ascomycete Yeast Candida lusitaniae
Source: PLoS One. 2014 Dec 8;9(12):e114531. doi: 10.1371/journal.pone.0114531 (PMC4259357; doi:10.1371/journal.pone.0114531)

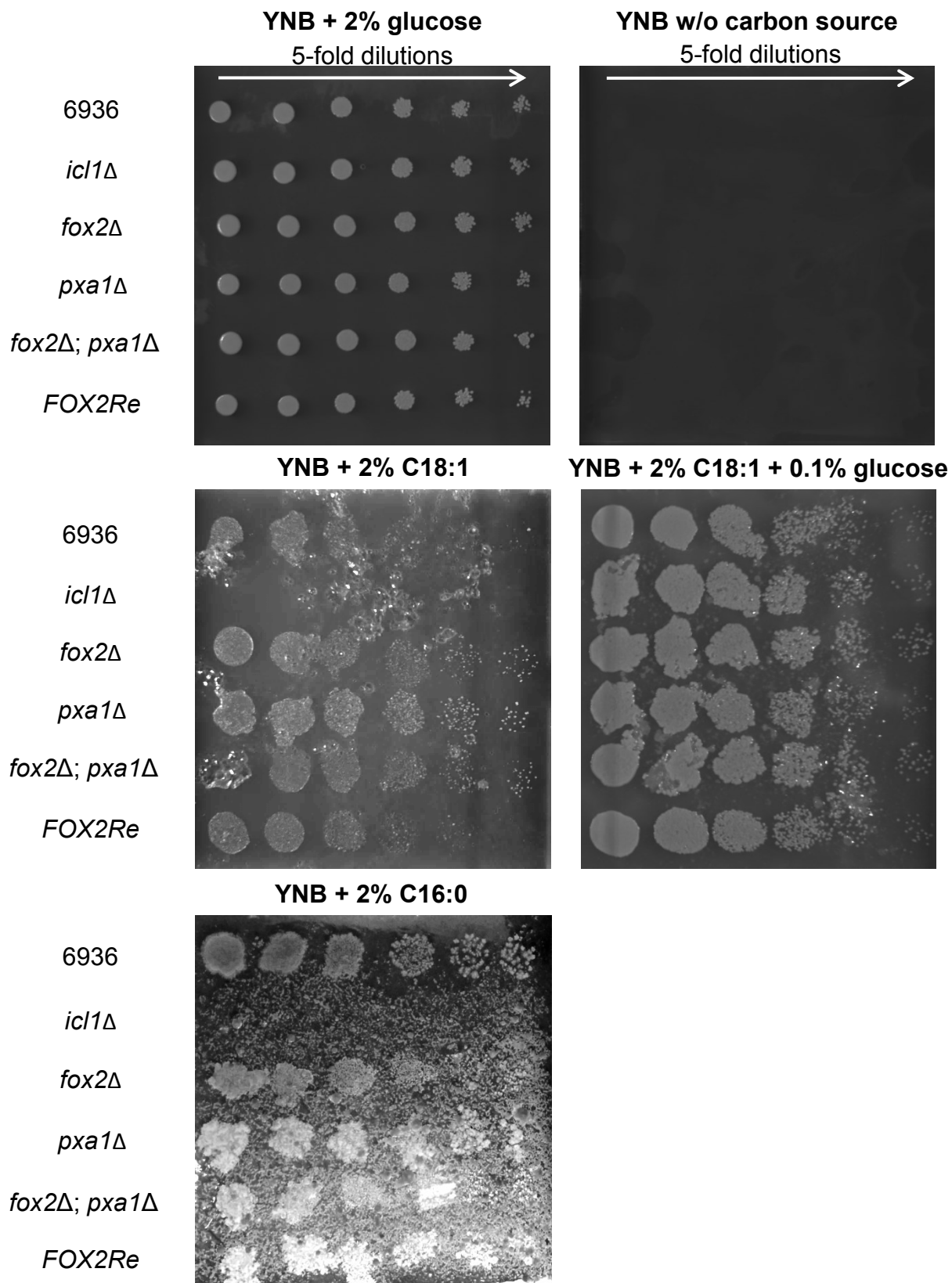

Figure S2.

Supplement: S2 Figure — Growth tests of wild-type and mutant strains of C. lusitaniae on YNB agar supplemented or not with different carbon sources. (PDF) [file pone.0114531.s002.pdf]

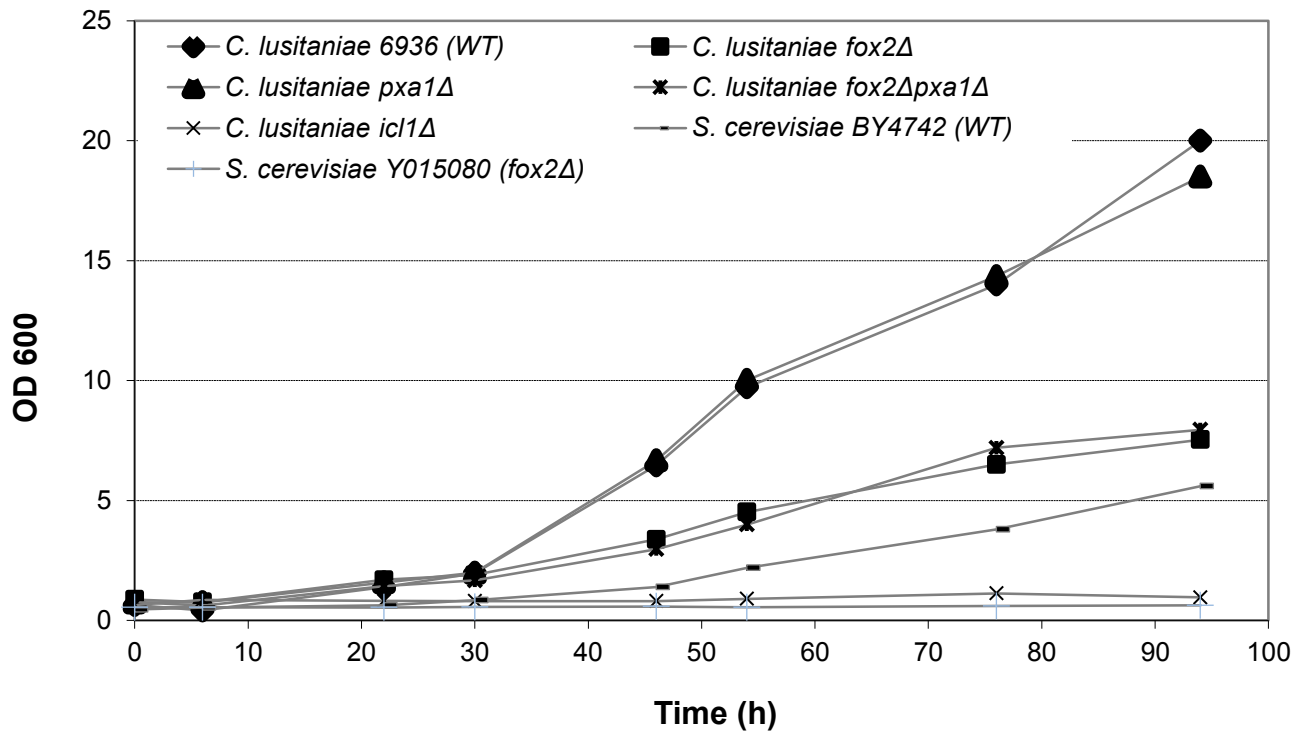

Figure S3.

Supplement: S3 Figure — Growth of C. lusitaniae and S. cerevisiae wild type and mutant strains in liquid YNB + 2% (v/v) C18:1. OD: optical density, WT: wild-type. For growth of the S. cerevisiae Y15080 fox2 strain, the medium was supplemented with 25 µg/ml of lysine, uracil, leucine and histidine. (PDF) [file pone.0114531.s003.pdf]
