# Supplementary material for: A Fox2-Dependent Fatty Acid ß-Oxidation Pathway Coexists Both in Peroxisomes and Mitochondria of the Ascomycete Yeast Candida lusitaniae
Source: PLoS One. 2014 Dec 8;9(12):e114531. doi: 10.1371/journal.pone.0114531 (PMC4259357; doi:10.1371/journal.pone.0114531)
Supplement: S1 Table — Genotypes of the Candida lusitaniae strains used in this study. (PDF) [file pone.0114531.s004.pdf]

Table S1. Genotypes of the *Candida lusitaniae* strains used in this study

Strain	Genotype	Source or reference
6936	*MAT* **a**, *URA3* , *ICL1* , *FOX2, PXA1* (wild-type)	CBS (Centraalbureau voor Schimmelcultures)
ura3 [Δ360]	*MAT* **a**, *ura3* [Δ360], *ICL1* , *FOX2, PXA1*	(26, 28)
ura3 [Δ990]	*MAT* **a**, *ura3* [Δ990], *ICL1* , *FOX2, PXA1*	(28)
icl1 Δ::GUN	*MAT* **a**, *icl1* Δ::*URA3, ura3* [Δ360], *FOX2, PXA1*	This study
icl1 Δ, *ura3* Δ	*MAT* **a**, *icl1* Δ, *ura3* [Δ360], *FOX2, PXA1*	This study
icl1 Δ	*MAT* **a**, *icl1* Δ, *URA3, FOX2, PXA1*	This study
ICL1Re	*MAT* **a**, *icl1* Δ::*ICL1 -URA3, ura3* [Δ360], *FOX2, PXA1*	This study
fox2 Δ::GUN	*MAT* **a**, *fox2* Δ::*URA3, ura3* [Δ360], *ICL1, PXA1*	This study
fox2 Δ, *ura3* Δ	*MAT* **a**, *fox2* Δ, *ura3* [Δ360], *ICL1, PXA1*	This study
fox2 Δ	*MAT* **a**, *fox2Δ, URA3, ICL1, PXA1*	This study
FOX2Re	*MAT* **a**, *fox2* Δ::*FOX2 -URA3, ura3* [Δ360], *ICL1, PXA1*	This study
pxa1 Δ	*MAT* **a**, *pxa1* Δ*::pGEMT-URA3-pxa1* [core], *ura3* [Δ990], *ICL1, FOX2*	This study
