# Supplementary material for: A Fox2-Dependent Fatty Acid ß-Oxidation Pathway Coexists Both in Peroxisomes and Mitochondria of the Ascomycete Yeast Candida lusitaniae
Source: PLoS One. 2014 Dec 8;9(12):e114531. doi: 10.1371/journal.pone.0114531 (PMC4259357; doi:10.1371/journal.pone.0114531)
Supplement: S2 Table — Oligonucleotides used in this study. (PDF) [file pone.0114531.s005.pdf]

Table S2. Oligonucleotides used in this study

Primer	Sequence (5' to 3')	Use
**NotURA3**	GACATGCAT**GCGGCCGC**ACAGAGGAGTAAGACAGG	Cloning *URA3* in complementation plasmids, *URA3* probe
**SpeURA3**	GCATCAC**ACTAGT**ACCCGTTGATGGCAGAGTTG	Cloning *URA3* in complementation plasmids, *URA3* probe
**BclGUN**	TCGAGA**TGATCA**CCCGAGGTCGCATGCTCC	Cloning *URA3* within *ICL1* ORF
**StuGUN**	CACGTG**AGGCCT**CCAAGCTATTTAGGTGACAC	Cloning *URA3* within *ICL1* ORF
**MfeGUN**	TCGAGA**CAATTG**CCCGAGGTCGCATGCTCC	Cloning *URA3* within *FOX2* ORF
**Bgl2GUN**	CGCGCG**AGATCT**CCAAGCTATTTAGGTGAC	Cloning *URA3* within *FOX2* ORF
**3'URA3F**	CTTCTCTATCCGTCTCTGTTCC	PCR characterization of transformants
**5'URA3R**	ACATCATCCTTCACCAACTCTGC	PCR characterization of transformants
**URA3del**	ACCGGTGACTCCATGAGCGTTG	PCR characterization of transformants
**URA3ex**	CTCAGCGTCAGGTGTTTACG	PCR characterization of transformants
**FICL1**	CGGTACATTACCTGTGCTTGC	Cloning *ICL1* in pGEM-T, *ICL1* probe
**RICL1**	GAGCAGTTCAGGTAAATCCG	Cloning *ICL1* in pGEM-T, *ICL1* probe
**FICL1ex**	ACTTCCAGATGGCTTCTCCA	PCR characterization of *icl1* transformants
**RICL1ex**	TTCAGTCCACATGGATGGTC	PCR characterization of *icl1* transformants
**FFOX2**	CGAGGTGTCACCATATAAGCC	Cloning *FOX2* in pGEM-T, *FOX2* probe
**RFOX2**	GCTCACGAACGAAAGCCTAC	Cloning *FOX2* in pGEM-T, *FOX2* probe
**FFOX2ex**	ATCTCGGTCCGTCATGAGTG	PCR characterization of *fox2* transformants
**RFOX2ex**	ATATGGCCTCCGAACAGAG	PCR characterization of *fox2* transformants
**5'Pxa1NcoI**	CAT**GCCATGGC**ACCAAGACGAAGGCGAG	Cloning *PXA1* [core] in pG-URA3, *PXA1* probe
**3'Pxa1SacII**	TCA**CCGCGG**GCTCAACATATCGTCGTACG	Cloning *PXA1* [core] in pG-URA3, *PXA1* probe
**5'Pxa1ex**	GACAGGTGCTATTGTTGGC	PCR characterization of *pxa1* transformants
**3'Pxa1ex**	CGTATCCCCTTTGATCTCCAACAC	PCR characterization of *pxa1* transformants
**FpET28NdeFox2**	GCCGCGCGGCAGC**CATATG**ATGACTGTCTCCTTCAAGGATAAGG	Cloning *FOX2* in pET28 for protein expression in *E. coli*
**RpET28XhoFox2**	GGTGGTGGTG**CTCGAG**CTACAACTTGCCCTTGTCACCAAT	Cloning *FOX2* in pET28 for protein expression in *E. coli*

Sequences in bold character are recognition sites for the restriction endonuclease specified in the name of the primer
