# Supplementary material for: A Fox2-Dependent Fatty Acid ß-Oxidation Pathway Coexists Both in Peroxisomes and Mitochondria of the Ascomycete Yeast Candida lusitaniae
Source: PLoS One. 2014 Dec 8;9(12):e114531. doi: 10.1371/journal.pone.0114531 (PMC4259357; doi:10.1371/journal.pone.0114531)
Supplement: S1 File — Identification of the ICL1 , FOX2 and PXA1 genes in the genome of C. lusitaniae . (DOCX) [file pone.0114531.s006.docx]

**File S1**

**Identification of the *ICL1*, *FOX2* and *PXA1* genes in the genome of *C. lusitaniae***. The genes *ICL1* encoding the isocitrate lyase*, FOX2* encoding the multifunctional protein of β-oxidation*,* and *PXA1* encoding part of an ABC transporter responsible for peroxisomal long chain FA uptake*,* were identified in the genome of *C. lusitaniae* (http://www.broadinstitute.org/) with a BLAST analysis [1] using as query the orthologous proteins of *C. albicans* (*Candida* Genome Database, http://www.candidagenome.org/). This *in silico* analysis allowed us to identify: **i/** the 1650-bp ORF CLUG_01411.1 encoding a predicted protein of 549 amino acids (61 kDa) having 79% identity and 89% similarity with Icl1p (orf19.6844) of *C. albicans*, **ii/** the 2709-bp ORF CLUG_01348.1 encoding a predicted protein of 902 amino acids (98 kDa) having 71% identity and 85% similarity with Fox2p (orf19.1288) of *C. albicans*, **iii/** the 2430-bp ORF CLUG_01238.1 encoding a predicted protein of 809 amino acids having 66% identity and 79% similarity with Pxa1p (orf19.7500) of *C. albicans*. The nucleotide sequences of *ICL1*, *FOX2* and *PXA1* were determined for the strain CBS6936 and were deposited in GenBank under the accession number JQ710936, JQ710937, and JQ710938, respectively. The *C. lusitaniae* Icl1p putative protein showed conservation of the K208, K209, C210, H212 amino acids, which were previously identified as the catalytic residues in the isocitrate lyase of *Escherichia coli* [2]. As in other yeasts, the *C. lusitaniae* Fox2p contains two dehydrogenase domains (DHaseA at aa 24-180, and DHaseB at aa 323-485), which are shortchain alcohol dehydrogenase/reductase superfamily members, and one enoyl-CoA hydratase domain (aa 780-895) [3]. Both Icl1p and Fox2p of *C. lusitaniae* contain a type I peroxisomal targeting signal (-GKL) at the COOH extremity of the protein [4], [5]. Synteny was conserved in the close vicinity of CLUG_01411.1 and CLUG_01348.1 in *C. lusitaniae* and in *C. albicans*, which reinforced the idea that these ORF are the true orthologs of the *ICL1* gene and *FOX2* gene of *C. albicans*. Furthermore, other paralog genes having significant homology with *ICL1* and *FOX2* could not be detected in the genome of *C. lusitaniae*. Pxa1p belongs to the superfamily of ATP-binding cassette (ABC) proteins, and to the adrenoleukodystrophy protein (ALDp) subfamily [6]. The *C. lusitaniae* Pxa1p putative protein have highly conserved residues corresponding to the Walker A (aa 566–589) and Walker B (aa 687-734) domains, possess an ABC signature (aa 668-716) and a motif that resembles (aa 603-635) a sequence described as “new motif” for the ALDp subfamily [7]. Synteny was partially conserved in the close vicinity of Pxa1p in *C. lusitaniae* and in *C. albicans.*

**Result S1 references**

1. Altschul S, Madden T, Schaffer A, Zhang J, Zhang Z, et al. (1997) Gapped BLAST and PSI-BLAST: a new generation of protein database search programs. Nucl Acids Res 25: 3389–3402.

2. Rehman A, McFadden BA (1997) Lysine 194 is functional in isocitrate lyase from *Escherichia coli*. Curr Microbiol 35: 14–17.

3. Ylianttila MS, Pursiainen NV, Haapalainen AM, Juffer AH, Poirier Y, et al. (2006) Crystal structure of yeast peroxisomal multifunctional enzyme: structural basis for substrate specificity of (3R)-hydroxyacyl-CoA dehydrogenase units. J Mol Biol 358: 1286–1295.

4. Gould SG, Keller GA, Subramani S (1987) Identification of a peroxisomal targeting signal at the carboxy terminus of firefly luciferase. J Cell Biol 105: 2923–2931.

5. Neuberger G, Maurer-Stroh S, Eisenhaber B, Hartig A, Eisenhaber F (2003) Prediction of Peroxisomal Targeting Signal 1 containing proteins from amino acid sequence. J Mol Biol 328: 581–592.

6. Shani N, Watkins PA, Valle D (1995) *PXA1*, a possible *Saccharomyces cerevisiae* ortholog of the human adrenoleukodystrophy gene. Proc Natl Acad Sci U S A 92: 6012 –6016.

7. Gaur M, Choudhury D, Prasad R (2005) Complete inventory of ABC proteins in human pathogenic yeast, *Candida albicans*. J Mol Microbiol Biotechnol 9: 3–15.
